# Global Gene-expression Analysis of the Response of *Salmonella* Enteritidis to Egg White Exposure Reveals Multiple Egg White-imposed Stress Responses

**DOI:** 10.3389/fmicb.2017.00829

**Published:** 2017-05-12

**Authors:** Florence Baron, Sylvie Bonnassie, Mariah Alabdeh, Marie-Françoise Cochet, Françoise Nau, Catherine Guérin-Dubiard, Michel Gautier, Simon C. Andrews, Sophie Jan

**Affiliations:** ^1^Agrocampus Ouest, UMR1253 Science et Technologie du Lait et de l'OeufRennes, France; ^2^INRA, UMR1253 Science et Technologie du Lait et de l'OeufRennes, France; ^3^Science de la Vie et de la Terre, Université de Rennes IRennes, France; ^4^School of Biological Sciences, University of ReadingReading, UK

**Keywords:** egg white, *Salmonella* Enteritidis, transcriptomic, envelop stress, iron

## Abstract

Chicken egg white protects the embryo from bacterial invaders by presenting an assortment of antagonistic activities that combine together to both kill and inhibit growth. The key features of the egg white anti-bacterial system are iron restriction, high pH, antibacterial peptides and proteins, and viscosity. *Salmonella enterica* serovar Enteritidis is the major pathogen responsible for egg-borne infection in humans, which is partly explained by its exceptional capacity for survival under the harsh conditions encountered within egg white. However, at temperatures up to 42°C, egg white exerts a much stronger bactericidal effect on *S*. Enteritidis than at lower temperatures, although the mechanism of egg white-induced killing is only partly understood. Here, for the first time, the impact of exposure of *S*. Enteritidis to egg white under bactericidal conditions (45°C) is explored by global-expression analysis. A large-scale (18.7% of genome) shift in transcription is revealed suggesting major changes in specific aspects of *S*. Enteritidis physiology: induction of egg white related stress-responses (envelope damage, exposure to heat and alkalinity, and translation shutdown); shift in energy metabolism from respiration to fermentation; and enhanced micronutrient provision (due to iron and biotin restriction). Little evidence of DNA damage or redox stress was obtained. Instead, data are consistent with envelope damage resulting in cell death by lysis. A surprise was the high degree of induction of hexonate/hexuronate utilization genes, despite no evidence indicating the presence of these substrates in egg white.

## Introduction

Avian egg white is an intracellular fluid that serves the dual purposes of protecting the developing embryo against invading microorganisms and providing it with a source of nutrients. Egg white represents a hostile medium for bacterial propagation due to its harsh physicochemical properties (alkaline pH and high viscosity), the nutritional restriction it imposes and its arsenal of antimicrobial molecules. Various macromolecules within egg white exhibit antimicrobial activity (Baron et al., 2016 for a review), including: lysozyme, which exerts a hydrolytic activity against the cell wall of Gram-positive bacteria leading to membrane disruption; ovotransferrin, a high-affinity iron-chelating protein that promotes iron restriction and mediates damage to bacterial cytoplasmic membranes; protease inhibitors (e.g., ovomucoid, ovoinhibitor, cystatin, and ovostatin) that inhibit proteases of pathogenic bacteria required for host colonization; and vitamin-binding proteins (flavoprotein, avidin, and the thiamine-binding protein) which sequester riboflavin, biotin, and thiamine, respectively, and thus induce bacteriostasis. In addition, some minor proteins and peptides recently revealed by high-throughput approaches may also play a role in defense against bacterial contamination and it is quite possible that the various anti-bacterial factors associated with egg white interact synergistically to enhance protection against bacterial invaders (Baron et al., 2016).

Studies on the antimicrobial activity of egg white generally employ *Salmonella enterica* serovar Enteritidis as the model bacterium as it represents the predominant (90%) serotype responsible for foodborne diseases (salmonellosis) resulting from egg or egg-product consumption (EFSA BIOHAZ Panel, 2014). Moreover, egg products (whole, yolk, or liquid egg white) are used in the fabrication of various foodstuffs (sausages, sauces, cakes, pasta, etc.) and it is clearly important that such egg products are pathogen-free, especially when preparation does not include cooking. However, the traditional heat treatment of liquid egg white (e.g., 57°C for just 2 min) does not result in the total destruction of *S*. Enteritidis, although does preserve the techno-functional properties of egg white (Baron, 2010). The high occurrence of the *S*. Enteritidis in egg-related food-borne disease can also be explained by the specialized ability of this serovar to survive under the harsh conditions encountered in egg white (Lock and Board, 1992; Clavijo et al., 2006; Guan et al., 2006; Gantois et al., 2008b, 2009a), although the mechanisms associated with the exceptional egg white resistance exhibited by *S*. Enteritidis have yet to be entirely resolved.

Many studies have focused on identifying *S*. Enteritidis factors conferring resistance to egg white. Approaches employed include directed mutagenesis (Lu et al., 2003; Cogan et al., 2004; Kang et al., 2006), random mutagenesis (Clavijo et al., 2006), *in vivo* expression technology (IVET) (Gantois et al., 2008a) and microarray-based transposon library screening (Raspoet et al., 2014). Such studies have revealed genes essential for the survival of *S*. Enteritidis in egg white, including those with roles in membrane structure and function, the metabolism of nucleic acids and amino acids, motility, the synthesis and repair of DNA, invasion and pathogenicity. However, comparison between such studies is complicated by the wide range of experimental conditions and methods employed, such that the relative contributions of individual components remains uncertain. Nevertheless, it is generally accepted that the key anti-bacterial influences of egg white are iron deficiency (provoking bacteriostatisis) and bacterial-cell envelop damage (which is bactericidal) (Kang et al., 2006). However, physico-chemical factors, such as alkaline pH and temperature of incubation, also play important roles in egg white antimicrobial activity. Immediately after laying, the loss of carbon dioxide through the pores of the eggshell leads to a rapid increase in egg white pH, from 7.8 to 9.3 over 4–14 days, depending on temperature (Sauveur, 1988). This increased alkalinity is important since, at pH ≥ 8.8, egg white displays bacteriostatic and/or bactericidal properties, whereas at pH 7.5 or 8 either weak bacterial growth or a bacteriostatic activity are observed, respectively (Tranter and Board, 1984; Messens et al., 2004; Kang et al., 2006).

Temperature also has a major impact on *S*. Enteritidis growth in egg white. At 20 and 30°C, many reports suggest that *S*. Enteritidis is able to grow slightly in egg white. The increase varies from one to four logarithmic units/mL depending on the authors and incubation time (Clay and Board, 1991; Lock and Board, 1992; Humphrey and Whitehead, 1993; Ruzicková, 1994; Baron et al., 1997; Chen et al., 2005; Murase et al., 2005). At 37°C, the results observed by different authors vary: several report a maintenance or death of *S*. Enteritidis in egg white (Bradshaw et al., 1990; Ruzicková, 1994; Clavijo et al., 2006; Guan et al., 2006) whilst others indicate a slight growth (Kang et al., 2006). Kang et al. (2006) suggest that growth, maintenance or death of *S*. Enteritidis incubated at 37°C in egg white depends on the inoculum size. However at 42°C, a bactericidal effect of egg white against *S*. Enteritidis is systematically observed, with destruction varying from <2 to 3.5 logarithmic units/mL, depending on incubation time and laboratory (Guan et al., 2006; Kang et al., 2006; Gantois et al., 2008a). The literature thus indicates that higher temperature and alkaline pH enhance the antibacterial activity of egg white. It is notable that temperatures that support the bactericidal activity of egg white are close to those encountered naturally during egg formation and that of the hen body (42°C); this temperature is routinely used in studies on the bactericidal activity of egg white (Guan et al., 2006; De Vylder et al., 2013; Raspoet et al., 2014). Indeed, the relation with temperature and antimicrobial activity of egg white is underlined by a patent describing a novel egg white pasteurization process (Liot and Anza, 1996) involving incubation of liquid egg white at moderate temperatures (best results at 42–45°C) for 1–5 days. This method is considered superior to the less efficient, but more traditional, “egg white pasteurization” treatment (e.g., 57°C for 2–5 min) since it provides a complete killing of *S*. Enteritidis, preserves the techno-functional properties of the egg white and allows subsequent storage of liquid egg white at room temperature rather than under refrigeration (Baron, 2010). Studying the global response of *S*. Enteritidis to egg white incubation at 42–45°C would provide insight into the killing mechanisms involved that might enable a further optimization of the control of this pathogen in eggs and egg products.

Recently, the contribution of temperature (37–48°C), pH (7.8 and 9.3), inoculum size (3–8 log_10_ CFU) and egg white-protein concentration (using egg white and egg white model medium) to the antimicrobial activity of egg white was more thoroughly investigated using factorial design analysis (Alabdeh et al., 2011). The results provided two major conclusions: firstly, that the key role played by egg white proteins in antimicrobial activity depends on both temperature and pH; and secondly, that the bactericidal activity of egg white against *S*. Enteritidis only becomes apparent at ≥42°C. These findings thus identify the conditions required for egg white dependent killing of *S*. Enteritidis allowing further research on the mechanisms involved. The study described here further addresses the bactericidal activity of egg white and the corresponding response of *S*. Enteritidis. We examine the global expression response of *S*. Enteritidis to egg white model medium exposure for 45 min at 45°C and pH 9.3, and reveal major changes in transcription that correlate well with the conditions presented by egg white. The results provide novel insight into the environmental signals encountered by *S*. Enteritis in egg white and the expression-response that this pathogen displays in its attempt to adapt to such exposure.

## Materials and methods

### Bacterial strain

*Salmonella* Enteritidis NCTC13349 was used in this study (kindly donated by Matthew McCusker, Center for Food Safety and Food Borne Zoonomics, Veterinary Sciences Centre, University College Dublin, Ireland). This strain was isolated from an outbreak of human food poisoning in the United Kingdom that was traced back to a poultry farm. The stock cultures were conserved at −20°C with 50% (v/v) glycerol. Before use, cells were propagated twice overnight at 37°C in tryptone soy broth (TSB, Merck, Darmstadt, Germany) without shaking.

### Preparation of sterile egg white and egg white model medium

Egg white was prepared from 5 to 10 day-old eggs obtained from conventional hen housing system. Eggshell surfaces were cleaned with tissue paper, checked for cracks and then sterilized with 70% alcohol; residual alcohol was removed by briefly flaming the shell. Eggshells were then broken, under sterile conditions, and the released egg whites were aseptically homogenized with a DI25 Basic homogenizer (Ika, Grosseron, Saint-Herblain, France) at 9,500 rpm for 1 min. The egg white pH was 9.3 ± 0.1.

Egg white filtrate was prepared by ultrafiltration of three different batches of liquid egg white (from different eggs). Ultrafiltration was performed using a pilot unit (TIA, Bollène, France) equipped with an Osmonics membrane (5.57 m^2^, 10 kDa cut-off; PW 2520F, Lenntech B.V., Delft, Netherlands). Filtration was achieved according to Baron et al. (1997). Concentrated egg white (retentate) was circulated back to the feedtank and permeate (filtrate) was drained off, collected in a beaker, sterilized by filtration (Nalgene® filter unit, pore size <0.2 μm, Osi, Elancourt, France), and then stored at 4°C until use. The pH (9.3 ± 0.1) of the egg white filtrate remained unchanged.

Egg white model medium (EWMM) was prepared by adding 10% egg white (vol/vol) to egg white filtrate. The solution was then homogenized with a DI25 basic homogenizer at 9,500 rpm for 1 min following which the pH was re-confirmed (9.3 ± 0.1). Medium sterility was monitored by inoculating tryptone soy agar (TSA, Merck, Darmstadt, Germany) plates with 1 ml of EWMM and then confirming lack of colony formation after overnight incubation at 37°C. The egg white filtrate pH was 9.3 ± 0.1.

### Incubation of *S*. enteritidis in egg white or egg white model medium

After propagation in tryptone soy broth, bacterial suspensions were centrifuged at 5,600 × g and 15°C for 7 min, and cells were washed three times with egg white filtrate. The final pellet was resuspended in the original volume of egg white filtrate and was then inoculated into egg white or in EWMM at a final concentration of 7.3 ± 0.2 log_10_ CFU/ml. The resuspended bacteria were incubated at 30 or 45°C for 45 min and 24 h to evaluate the bactericidal activity of egg white and EWMM. Bacterial suspensions of *S*. Enteritidis (overnight cultures centrifuged at 5,600 × g and 15°C for 7 min, and washed three times with fresh optimum medium, TSB) were also incubated in TSB at pH 7.3 for 24 h at 30 and 45°C, as a control.

For transcriptome analysis, bacterial suspensions of *S*. Enteritidis prepared as above were incubated in EWMM (7.3 ± 0.2 log_10_ CFU/ml in aliquots of 20 ml) for 7, 25, or 45 min at 45°C before RNA extraction.

### Numeration of bacterial cells after incubation

A numeration method based on miniaturization of the conventional plate-counting technique was used according to Baron et al. (2006) with a Tryptone soya agar (Merck, Darmstadt, Germany) overlay procedure. After incubation at 37°C for 20–24 h, the number of colony forming units (CFU) was recorded. Results were compared using analysis of variance and the average comparison test using the R 2.13.0 software (http://cran.r-project.org/).

### RNA isolation and synthesis of cDNA

After incubation, cells were centrifuged at 10,000 × g at 4°C for 5 min, pellets were immediately frozen in liquid nitrogen and stored at −80°C. After defrosting on ice, cells were disrupted by treatment with TE (10 mM Tris-HCl pH 7, 1 mM EDTA) buffer, containing 20 mg/mL lysozyme, for 30 min at 37°C followed by mechanical lysis with zirconium beads using a FastPrep-24 instrument (MP Biomedicals, Illkirch, France) with two cycles of 30 s at 30 Hz interspaced with 30 s cooling periods. Total RNA was then isolated by phenol-chloroform extraction. The evaluation of RNA quantity and quality was assessed spectrophotometrically by measuring the ultraviolet absorbance profile (NanoDrop, NanoDrop Technologies, Inc., Rockland, Wilmington, DE, USA) at 230, 260, and 280 nm. For the microarray experiment, additional analysis for RNA integrity was performed using a RNA 6000 Nano LabChip kit (2100 Bioanalyzer, Agilent Technologies, Santa Clara, CA, USA) (Mueller et al., 2000). The RNA samples were then DNase treated using a DNA-free kit (Ambion, Austin, TX, USA) according to the manufacturer's instructions. Quantification of the RNA and any contamination by proteins was again assessed using a NanoDrop ND-1000 and RNA integrity again confirmed using a 2100 Bioanalyzer. The resultant total RNA (500 ng of each sample) was reverse transcribed and labeled using the SuperScriptTM Indirect cDNA Labeling System (Invitrogen, Life Technologies, Courtaboeuf, France) according to the protocol provided by the manufacturer, except that the hexamer solution was replaced with the pdN6 hexamer solution (Roche Diagnostics, Meylan, France).

### Microarray and experimental design

A DNA microarray was designed based on the only published genome sequence for *Salmonella* Enteritidis strain NCTC13349 (http://www.ncbi.nlm.nih.gov/nuccore/AM933172.1). The microarray, containing 3971 ORFs (representing 94.4% of *S*. Enteritidis gene composition), was designed using Agilent's e-array software (https://earray.chem.agilent.com/earray/). Note that 235 ORFs were not included due to restricted design parameters of the e-array program. The custom oligonucleotide microarray was manufactured by Agilent Technologies using an 8 × 15K format and included each probe in duplicate. Microarray hybridization and data analysis were performed by the Pasteur Institute (Transcriptome and Epigenome Platform PF2, Paris, France).

*S*. Enteritidis, exposed to egg white model medium for 7, 25, and 45 min at 45°C, was compared to a reference control (0 min of incubation) consisting of *S*. Enteritidis grown overnight in TSB at 37°C, washed three times in egg white filtrate and finally resuspended in egg white filtrate at ambient temperature. For each time point, three arrays were hybridized with three independent biological replicates in duplicate (giving three biological replicates and two technical replicates). Cy3 and Cy5 dye-swap design was included in order to reduce dye-specific effects. The data have been deposited in NCBI's Gene Expression Omnibus and are accessible through GEO Series accession number GSE92545 (http://www.ncbi.nlm.nih.gov/geo/query/acc.cgi?acc=GSE92545).

### Data acquisition and preprocessing

Images of the microarrays were scanned using an Axon 4000a scanner (Axon, Instruments, CA, USA) and intensity data were extracted using the GenepixPro 6.1 software. Raw microarray data were first normalized using the LOWESS (Locally Weighted Scatter Plot Smoother) regression option in the R software suite, version 2.10.1 (http://www.r-project.org/), to correct for dye-bias within the array, followed by median normalization to normalize across all arrays.

### Differential expression analysis

Changes in gene expression upon incubation in EWMM compared to the control were recorded as fold changes (ratio of 7, 25, or 45–0 min). Statistical significance was estimated with a moderated *t-test* using the LIMMA package (Smyth, 2004) of the R software suite (version 2.10.1). The adjustment of raw *p*-values was then performed to account for multiple comparisons, using the method of Benjamini and Yekutieli (2001). Genes that exhibited ≥2.0 fold changes in expression (with respect to the control) and *P* ≤ 0.05 were considered as differentially regulated. Differentially expressed genes were initially categorized by function according to the Clusters of Orthologous Groups (http://www.ncbi.nlm.nih.gov/COG) designations and categorization was subsequently optimized manually.

### Confirmation of selected genes by qRT-PCR analysis

Confirmation of the transcriptomic analyses was achieved using qRT-PCR. Primers (Table 1) for amplification of selected genes were designed using Primer 3 (http://primer3.ut.ee/). Non-contamination of RNA by gDNA was confirmed by qPCR prior to cDNA synthesis. cDNA was synthesized using the high-capacity cDNA archieve kit (Applied Biosystems) as recommended by the manufacturer. qRT- PCR was performed using an iCycler iQ Real-Time PCR Detection System (Biorad). Thermal cycling consisted of 5 min at 95°C, followed by 45 cycles of 15 s at 95°C, 20 s at 60°C and 40 s at 72°C. A melting curve analysis (55°C to 95°C) was performed after the thermal profile to ensure specificity and PCR efficiency was calculated at between 85 and 105% from the log-linear portion of the standard curves. A total of two RNA extractions were performed using distinct *S*. Enteritidis cultures grown on different batches of EWMM. Each of the two RNA extracts thus obtained was subject to qRT-PCR, in triplicate, for each selected gene. Standard curves were generated to calculate the copy number of each gene in each sample. The most stable control genes in our conditions were determined by geNorm among five potential genes. qRT-PCR data were normalized by geometric averaging of three internal control genes (*asmA, emrA*, orf32; primers in Table 1). Gene expression is thus provided as relative expression with respect to the normalization factor calculated by geNorm.

**Table 1:** **Sequences of primers used for qPCR**.

**Gene**	**Forward primer**	**Reverse primer**
*asmA*	ACCGGACACGTTCAGGTAAC	GGCAACAGGTTGTCCAGATT
*bioB*	CCGAGCGTTTAATGGAGGTA	TTGACGCCCTGTACAATCTG
*dgoK*	TTCTGATTGCCTGCTCTCCA	GCAATTGACTGGGGATCGAC
*emrA*	ATCTGTGGGTGGACGCTAAC	CCATATCCAGACCGACGACT
*fes*	CGCGTTTTGGCTGTGTACTA	TTCAGCCGGGTAATGACTTC
*ftn*	GCCACATACCACTGCAAGAA	CGACCTATGAGCATGAGCAG
*iroB*	TTTGTCGGTCCACCACTGTA	AGCGTCAAATACCACCAACC
*orf32*	CGGCTCTTTAACGCTCTGAC	CCGGTGGGTTTTGATAAATG

## Results

### Egg white and egg white model medium exhibit strong bactericidal activity at 45°C, but are only bacteriostatic at 30°C

**Figure 1:**
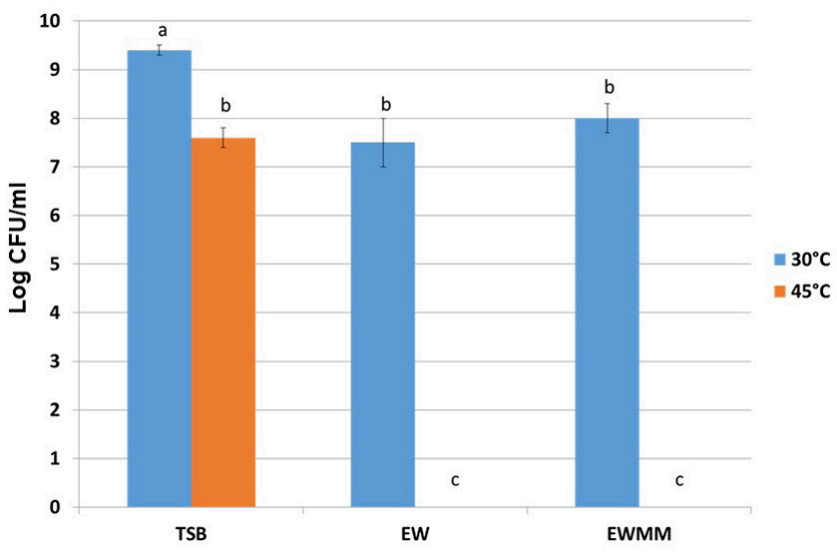
*****S***. Enteritidis survival after 24 h incubation at 30 or 45°C in optimum medium (TSB), egg white (EW) or in egg white model medium (EWMM)**. Inoculum levels were 7.3 ± 0.2 log_10_ CFU/ml. Experiments were performed in triplicate. Samples with different letters are significantly different (*p* < 0.05).

In order to establish suitable conditions for investigating the impact of egg white bacteriocidal activity on *S*. Enteritidis global gene expression, the effects of egg white (EW) and egg white model medium (EWMM; egg white filtrate supplemented with 10% egg white) on growth and survival of *S*. Enteritidis after 24 h were examined at both 30 and 45°C, at pH 9.3 (which corresponds to the natural pH of egg white, as achieved a few days after laying). Pre-cultures in tryptone soya broth (TSB, pH 7.3) were washed (three times) in EW filtrate and inoculated into EW and EWMM to give 7.3 ± 0.2 log_10_ CFU/ml. At 30°C, there was only slight growth (increases of ~0.2 and 0.7 log_10_ CFU/ml, respectively) whereas when a rich (TSB) medium was employed good growth (increase of 2.1 log_10_ CFU/ml) was achieved after 24 h at 30°C (Figure 1). These observations confirm that both EW and EWMM impose a bacteriostatic influence on *S*. Enteritidis at 30°C (Baron et al., 1997). In contrast, after incubation at 45°C for 24 h (Figure 1), *S*. Enteritidis was undetectable in either EW or EWMM (indicating a reduction of 7.3 log_10_ CFU/ml), while in TSB at 45°C slight growth was observed (increase of 0.3 log_10_ CFU/ml). Thus, EW and EWMM both exhibit strong bactericidal activity at 45°C, but are only bacteriostatic at 30°C. The similarity between the effects of EW and EWMM, as observed above, were previously described (Baron et al., 1997; Alabdeh et al., 2011) and validate the use of EWMM in place of EW in the gene expression studies described below. The use of EWMM in place of EW was necessary in order to enable easy recovery of *S*. Enteritidis following exposure to EWMM, which is not possible with EW due to its highly viscous consistency.

### Egg white exposure induces major changes in the *S*. enteritidis transcriptome

The above data show that EWMM elicites *S*. Enteritidis cell death at 45°C. Thus, incubation at 45°C in EWMM represents suitable conditions for examination of the response of *S*. Enteritidis to the bactericidal activity of EW using a global-expression analysis approach. For this purpose, relatively short incubation times (7, 25, and 45 min) were selected, where no major reduction in viable cell number occurred (observed change was from 7.3 ± 0.2 log_10_ to 7.1 ± 0.3 log_10_ CFU/ml at 0–45 min, respectively), which corresponds to the early phase of EWMM-induced lethal-cell damage. It should be noted that continued exposure resulted in progressive cell death (at a rate of ~1 log_10_ CFU/ml every 3 h; data not shown) leading to no detectable cells after 24 h of incubation (Figure 1). Expression effects were determined using a *Salmonella* microarray representing 94.4% of the genome (~3,971 genes). For each incubation time in EWMM, expression was compared to that of the control (*t* = 0: *S*. Enteritidis grown overnight at 37°C in TSB and then washed three times in egg white filtrate). Genes with a statistically significant ≥ twofold change in expression (*P* ≤ 0.05) were considered as differentially regulated. Thus, at 7, 25, and 45 min, 13.4% (288 induced and 277 repressed), 15.3% (304 induced and 362 repressed) and 18.7% (318 induced and 468 repressed) of genes were differentially regulated, respectively. This indicates that a high proportion of the genome was subject to expression alteration and that there was an increasing degree of change with time upon exposure to EWMM at 45°C. Examination of the expression data showed that the differentially-regulated genes can be classified into 11 major functional groups (Tables 2–**11**), and that these can be further organized into three broad functional categories: nutrient deprivation; cell damage/stress; and shift in energy metabolism and catabolism.

**Table 2:** **Biotin biosynthesis and utilization**.

**Gene**	**Function**	**Fold change**		
		**7 min**	**25 min**	**45 min**
**BIOTIN BIOSYNTHESIS**				
*bioA*	Adenosylmethionine-8-amino-7-oxononanoate transaminase	4.32	6.01	6.65
*bioB*	Biotin synthetase	12.31	19.50	23.39
*bioC*	Biotin biosynthesis protein BioC	5.89	13.22	16.55
*bioD*	Dithiobiotin synthetase	6.51	14.23	19.76
*bioF*	8-amino-7-oxononanoate synthase	9.75	19.11	25.41
**FATTY ACID METABOLISM**				
*accB*	Acetyl-CoA carboxylase biotin carboxyl carrier protein subunit	n.s.	3.31	4.75
*accC*	Acetyl-CoA carboxylase biotin carboxylase subunit	n.s.	3.43	5.81

*Highlighted fold changes correspond to up-regulated (dark gray) and down-regulated (light gray) genes. (n.s., non-significant), fold changes with p > 0.05*.

### Nutrient deprivation

#### Induction of genes involved in biotin biosynthesis

The genes of the *bioABCDF* operon, encoding the biotin biosynthesis components, were strongly up-regulated following 7–45 min exposure to EWMM at 45°C; expression was between 6.75- and 25.41-fold increased at 45 min (Table 2). This induction matches the poor biotin availability in egg white resulting from the presence of avidin, a powerful biotin-chelation protein (Banks et al., 1986), There was also a 4.75- to 5.81-fold increase in expression of the biotin-related *accBC* operon at 45 min. The *accB* gene encodes the Biotin Carboxyl Carrier Protein which is the direct recipient of biotin following synthesis and forms part of acetyl-CoA carboxylase that catalyzes the first step in fatty acid biosynthesis (Cronan, 1996; Streit and Entcheva, 2003; Beckett, 2007). Coordinated induction of *accBC* is necessary for stimulation of the synthesis of biotin (Abdel-Hamid and Cronan, 2007).

#### Induction of genes involved in iron-restriction response

A substantial expression effect was observed for genes involved in the response to iron starvation, with 49 genes in this functional category exhibiting significant expression changes. The overall differential expression of iron-starvation genes steadily increased over the time period of egg white exposure (0–45 min, Table 3), suggesting a sustained iron-restriction effect over this time. Under iron restriction, bacteria typically synthesize and secrete high-affinity ferric chelators, called siderophores, which solubilize exogenous iron, making it available for uptake (Neilands, 1995). *Salmonella* produces two types of cathecholate siderophore, enterobactin and salmochelin, but it can also pirate hydroxamate siderophores generated by competing microbes. The uptake of ferri-siderophore complexes involves specific outer-membrane (OM) receptors, an OM energy-transducing system and a periplasmic-binding protein dependent ATP-binding cassette inner-membrane permease (Andrews et al., 2003). The ferric uptake regulator (Fur) acts as the master regulator of iron homeostasis in the Enterobacteriaceae, controlling the iron-uptake machinery according to iron regime.

**Table 3:** **Iron starvation genes**.

**Gene**	**Function**	**Fold change**		
		**7 min**	**25 min**	**45 min**
**IRON ACQUISITION ENTEROBACTIN BIOSYNTHESIS**				
*entA*	2,3-dihydroxybenzoate-2,3-dehydrogenase	n.s.	4.35	4.96
*entB*	Isochorismatase	2.81	5.70	6.43
*entC*	Isochorismate synthase EntC	3.07	5.59	7.22
*entD*	Phosphopantetheinyltransferase component of enterobactin synthase multienzyme complex	n.s.	4.00	7.81
*entE*	Enterobactin synthase subunit E	2.60	5.35	5.83
*entF*	Enterobactin synthase subunit F	n.s.	4.40	5.67
*entH*	Thioesterase *(ybdB)*	n.s.	3.75	4.49
*entS*	Enterobactin exporter *(ybdA)*	2.63	3.02	4.13
**FERRIC-ENTEROBACTIN UPTAKE**				
*cirA*	Colicin I outer membrane receptor and translocator; ferric iron-catecholate transporter	2.76	3.71	4.01
*fepA*	Outer membrane receptor FepA	2.78	4.49	7.18
*fepB*	Iron-enterobactin transporter periplasmic binding protein	4.38	5.03	6.05
*fepC*	Iron-enterobactin transporter ATP-binding protein	3.23	3.95	5.63
*fepD*	Iron-enterobactin transporter membrane protein	2.97	3.60	4.52
*fepG*	Iron-enterobactin transporter permease	2.37	3.09	4.40
**FERRIC HYDROXAMATE UPTAKE**				
*fhuA*	Ferrichrome outer membrane transporter	3.65	3.86	4.57
*fhuB*	Iron-hydroxamate transporter permease subunit	n.s.	n.s.	1.77
*fhuC*	Iron-hydroxamate transporter ATP-binding subunit	n.s.	2.36	3.25
*fhuD*	Iron-hydroxamate transporter substrate-binding subunit	n.s.	2.33	3.35
*fhuE*	Ferric-rhodotorulic acid outer membrane transporter	3.39	3.92	3.75
*fhuF*	Ferric-iron reductase protein	6.04	7.43	10.13
**SALMOCHELINS BIOSYNTHESIS AND FERRIC SALMOCHELIN UPTAKE**				
*iroB*	Glycosyltransferase	n.s.	2.82	4.11
*iroC*	ABC transporter protein	n.s.	2.04	2.75
*iroD*	Ferric enterochelin esterase	n.s.	1.64	2.30
*iroE*	Hydrolase	n.s.	n.s.	1.59
*iroN*	Outer membrane receptor IroN	n.s.	2.25	2.47
**TonB-ExbB-ExbD ENERGY TRANSDUCTION SYSTEM**				
*exbB*	Biopolymer transport protein ExbB	2.86	2.98	3.19
*exbD*	Biopolymer transport protein ExbD	2.42	2.56	2.77
*tonB*	Transport protein TonB	4.06	4.47	4.95
**HYDROLYSIS OF ENTEROBACTIN AND FERRIC ENTEROBACTIN**				
*fes*	Enterobactin/ferric enterobactin esterase	7.08	10.01	11.91
**IRON STORAGE/RELEASE**				
*bfd*	Bfr-associated ferredoxin	2.32	2.37	2.09
*ftnA*	Ferritin *(ftn)*	n.s.	0.37	0.19
*yqjH*	Feric-siderophore reductase	2.72	2.04	2.02
**Fe-S CLUSTER ASSEMBLY DURING IRON STARVATION**				
*sufA*	Iron-sulfur cluster assembly scaffold protein	3.66	6.30	8.54
*sufB*	Cysteine desulfurase activator complex subunit SufB	3.23	6.49	9.35
*sufC*	Cysteine desulfurase ATPase component	n.s.	3.67	5.22
*sufD*	Cysteine desulfurase activator complex subunit SufD	n.s.	3.03	4.53
*sufS*	Bifunctional cysteine desulfurase/selenocysteine lyase	n.s.	2.55	3.77
**REPLACEMENT OF IRON ENZYMES WITH IRON-INDEPENDENT ALTERNATIVES DURING IRON STARVATION**				
*nrdE*	Ribonucleotide-diphosphate reductase subunit alpha	9.14	19.30	15.40
*nrdF*	Ribonucleotide-diphosphate reductase subunit beta	6.15	15.12	11.74
*nrdH*	Glutaredoxin-like protein	3.72	6.23	5.18
*nrdI*	Ribonucleotide reductase stimulatory protein	5.92	12.01	9.04
*sodA*	Superoxide dismutase	2.53	3.48	3.05
*sodB*	Superoxide dismutase	0.39	0.23	0.14
**MANGANESE UPTAKE**				
*mntH*	Fe/Mn transporter; NRAMP family	1.70	2.09	2.12
*sitA*	Fe/Mn transport protein, periplasmic-binding protein	5.31	7.19	9.54
*sitB*	Fe/Mn transport protein, ATP-binding component	4.32	6.03	8.51
*sitC*	Fe/Mn transport protein, inner membrane component	3.42	4.94	6.93
*sitD*	Fe/Mn transport protein, inner membrane component	2.27	3.47	4.71
**IRON UPTAKE FROM HEME**				
*ydiE*	Hemin uptake protein HemP	4.35	4.47	3.73

*Highlighted fold changes correspond to up-regulated (dark gray) and down-regulated (light gray) genes. (n.s.), fold changes with p > 0.05*.

Exposure of *Salmonella* to EWMM at 45°C stimulated a strong expression of the *entABCDEFHS* gene cluster (4.13- to 7.81-fold at 45 min) which encodes the proteins involved in biosynthesis and export of enterobactin, the major siderophore of *Salmonella*. The genes involved in ferric-enterobactin uptake and utilization (*fepABCDG, fes*, and *cirA*) were likewise strongly induced (4.01- to 7.18-fold at 45 min) as were the genes involved in ferric-hydroxamate uptake (*fhuABCD* and *fhuE*, 1.77 to 4.57 fold at 45 min) and ferric-hydroxamate utilization (*fhuF*, tenfold at 45 min). The *iro* genes, required for conversion of enterobactin to salmochelin as well as uptake and utilization of ferric-salmochelin, were also significantly induced (1.59- to 4.11-fold at 45 min) but to a lesser extent than the afore mentioned iron-uptake genes. The energy-transducing TonB-ExbB-ExbD system was also up-regulated (2.77- to 4.95-fold at 45 min). The Fe-Fur induced *ftnA* gene (encoding ferritin A, an iron-storage protein) was suitably repressed, an effect that increased from 7 to 45 min (0.19-fold at 45 min).

All the above genes are Fe-Fur regulated (Bjarnason et al., 2003; McHugh et al., 2003). The combined induction of the iron-acquisition genes and repression of the iron-storage gene strongly suggest that exposure of *Salmonella* to EWMM induces a Fe-Fur controlled homeostatic reaction in response to the prevailing iron-restriction conditions of the medium.

The Fur-mediated response of *Salmonella* was not limited to adjustment in iron uptake and storage capacity. The Fe-Fur repressed *sufABCDS* operon, encoding an alternative Fe-S cluster biosynthetic pathway employed during redox stress and iron starvation in *E. coli* (Outten et al., 2004), was also induced (3.77- to 9.35-fold at 45 min). In addition, the *nrdHIEF* genes specifying an alternative, Fe-Fur repressed, Mn-dependent ribonucleotide reductase (Martin and Imlay, 2011), were induced 5.18- to 15.4-fold at 45 min. The expression of the *sodA* and *sodB* genes, involved in the response to superoxide (redox-cycling agents) stress, was also significantly affected by EWMM exposure. SodA is a Mn-dependent superoxide dismutase whereas SodB is Fe-dependent. Both are Fe-Fur controlled, but in a reciprocal fashion—*sodA* is Fe-Fur repressed whilst *sodB* is Fe-Fur (via RfrA and RfrB) induced (Troxell et al., 2011). Upon exposure to EWMM, *sodA* was induced (3.05-fold at 45 min) and *sodB* was repressed (0.14-fold at 45 min). A similar pattern of expression was observed by Dubrac and Touati (2000, 2002) and McHugh et al. (2003).

The *sitABCD* operon, encoding a manganese-uptake system active in alkaline conditions (Kehres et al., 2002), was also induced (4.71- to 9.54-fold at 45 min) in EWMM. This system is known to be induced by low iron in response to Fur, but also by low Mn^2+^ in response to MntR (Ikeda et al., 2005). It has the capacity to import ferrous iron, but has strong preference for Mn^2+^ over Fe^2+^ (Kehres et al., 2002). Mn^2+^ uptake in *E. coli* only occurs when iron is depleted, probably to prevent interference of Mn^2+^ with cellular iron distribution (Anjem et al., 2009; Andrews, 2011). A similar effect would be anticipated for *Salmonella*. The Fur- and MntR-controlled *mntH* gene, also involved in Mn^2+^ and Fe^2+^ uptake (with a preference for Mn^2+^), was also induced but in lesser extent (up to 2.1-fold at 45 min). The greater induction response of *sitABCD* than that of *mntH* may be related to the distinct pH preferences of their encoded products; MntH exhibits optimal Mn^2+^ transport under acidic conditions whereas SitABCD has preference for alkaline pH (Kehres et al., 2002).

The *ydiE* gene was also induced by EWMM. This gene encodes a protein of unclear function and is related to the heme-utilization component, HemP of *Yersinia enterolitica* (Stojiljkovic and Hantke, 1992; Panina et al., 2001). The *ydiE* gene is Fe-Fur repressed in *S*. Typhimurium (Lutz and Bujard, 1997; Bjarnason et al., 2003), and its induction in EWMM is consistent with Fe-Fur repression in *S*. Enteritidis. The Fe-Fur repressed *yqjH* and *bfd* genes are considered to be involved in iron mobilization (Miethke et al., 2011; Yao et al., 2012). Appropriately, both were induced by EWMM (Table 3). Interestingly, there was no significant change in the expression of the *feoABC* operon, encoding the anaerobic, high-affinity ferrous-iron transporter. This may reflect the requirement for anoxic conditions for strong *feo* induction (Kammler et al., 1993).

In summary, the microarray data indicate a major iron-starvation response for *S*. Enteritidis.

#### Repression of genes involved in virulence

Fifteen genes among the ~30 genes located within *Salmonella* Pathogenicity Island 1 (SPI1) were significantly down regulated during the 45 min exposure to EWMM at 45°C (from 0.07- to 0.48-fold at 45 min; Table 4). SPI1 encodes a type III secretion system (T3SS) required for invasion of host gut epithelial cells in the early stages of infection. SPI1 expression is modulated by several environmental signals (Jones, 2005), which ensures its availability for invasion of epithelial cells when *Salmonella* reaches the distal small intestine. SPI1 is induced by iron in a Fur-dependent fashion (Teixidó et al., 2011) suggesting that the down regulation of the SPI1 genes observed here is due to the low iron concentration of egg white and consequent regulation change mediated by Fur.

**Table 4:** **SPI1 genes**.

**Gene**	**Function**	**Fold change**		
		**7 min**	**25 min**	**45 min**
*hilD*	AraC-family transcriptional regulator (SPI1)	0.36	0.20	0.16
*invA*	EscV/YscV/HrcV family type III secretion system export apparatus protein	0.34	0.25	0.17
*invB*	Surface presentation of antigens protein SpaK	n.s.	0.37	0.26
*invE*	Cell invasion protein (SPI1)	0.23	0.19	0.18
*invG*	Secretin EscC/YscC/HrcC family type III secretion system outer membrane ring	0.28	0.22	0.21
*invH*	Invasion lipoprotein InvH	0.12	0.07	0.07
*invI*	Surface presentation of antigens protein SpaM	n.s.	n.s.	0.30
*orgA*	Oxygen-regulated invasion protein OrgB	n.s.	0.30	0.23
*prgH*	Pathogenicity 1 island effector protein (SPI1)	0.30	0.22	0.19
*prgI*	EscF/YscF/HrpA family type III secretion system needle major subunit	0.32	0.24	0.19
*prgK*	EscJ/YscJ/HrcJ family type III secretion inner membrane ring protein	0.50	0.39	0.27
*sicA*	CesD/SycD/LcrH family type III secretion system chaperone	n.s.	n.s.	0.28
*sipB*	Cell invasion protein SipB	0.67	n.s.	0.48
*spaP*	EscR/YscR/HrcR family type III secretion system export apparatus protein	0.63	0.55	0.46
*sprB*	Transcriptional regulator	0.46	0.31	0.25

*Highlighted fold changes correspond to up-regulated (dark gray) and down-regulated (light gray) genes. (n.s.), fold changes with p > 0.05*.

### Cell damage/stress

#### Induction of genes involved in the envelope-stress response, mediated by CpxAR, OmpR/EnvZ and PspF regulators

A strong up-regulation of genes specifying proteins involved in various aspects of cell-envelop integrity was observed (Table 5). The induction of the *spy* gene increased with time of incubation giving a 22.18-fold increase at 45 min. The *spy* gene encodes a periplasmic chaperone protein that is induced by spheroplast formation, indole or zinc exposure as well as by misfolded envelope proteins (Hagenmaier et al., 1997; Yamamoto et al., 2008). Its expression is regulated by the two components systems, BaeSR and CpxAR (Raffa and Raivio, 2002), that mediate response to a variety of environmental stresses including suboptimal pH and factors perturbing the bacterial envelope (Raivio et al., 2013). Another CpxAR-regulated gene, *htrA* (*degP*), was also strongly induced (7.79-fold at 45 min). *degP* encodes a periplasmic, membrane-associated serine endoprotease that degrades abnormal proteins in the periplasm, preventing build-up of aggregated proteins.

**Table 5:** **Membrane-stress related gene**.

**Gene**	**Function**	**Fold change**			
		**7 min**	**25 min**	**45 min**	**Control by**
**CELL INTEGRITY**					
*htrA (degP)*	*htrA*; membrane-associated serine endoprotease, periplasmic	4.83	8.32	7.79	CpxAR [2, 9, 13, 18]
*spy*	Spheroplast protein Y, periplasmic	7.62	12.53	22.18	CpxAR, BaeSR [3, 4, 9, 11, 12, 18, 19]
*tolA*	Tolerance to colicins and phage; cell envelope integrity;	1.59	1.97	2.53	CpxAR [12], PspF [12]
*tolB*	Tolerance to colicins and phage; cell envelope integrity;	1.76	1.89	2.15	CpxAR[12], PspF [12]
*tolQ*	Tolerance to colicins and phage; cell envelope integrity	2.41	3.07	3.80	CpxAR[12]
*tolR*	Tolerance to colicins and phage; cell envelope integrity	2.30	2.86	3.62	
*ybgC*	Acyl-CoA thioester-hydrolase	2.27	2.68	3.31	CpxAR[12]
*yncJ*	Secreted hypothetical protein	6.35	14.31	19.35	CpxAR [18]
*yjfN*	Secreted hypothetical protein	n.s.	3.04	3.79	CpxAR [4, 18]
**CELL PERMEABILITY**					
*nmpC (ompD)*	Outer membrane porin protein	n.s.	0.38	0.32	CpxR [15, 18]
*ompC*	Outer membrane porin protein C	3.55	3.94	2.53	CpxAR [4, 6, 9, 13, 18], EnvZ/OmpR [10]
*ompF*	Outer membrane protein F prcursor	n.s.	n.s.	0.33	CpxAR [4, 6, 9, 13, 18], EnvZ/OmpR [10]
*ompS*	Outer membrane protein S1	n.s.	2.20	2.11	EnvZ/OmpR [17]
*ompX*	Outer membrane protein X	n.s.	2.27	2.19	EnvZ/OmpR [17]
**MULTIDRUG EFFLUX SYSTEM**					
*acrD*	Aminoglycoside/multidrug efflux system	1.96	2.42	3.60	CpxR, BaeSR [8, 9, 13, 14, 18]
*emrD*	Multidrug resistance protein D	1.98	2.47	3.34	EnvZ /OmpR [5]
**PEPTIDOGLYCAN METABOLISM**					
*amiC*	N-acetylmuramoyl-L-alanine amidase	2.74	2.87	3.05	CpxAR [13, 16]
*dacC*	D-alanyl-D-alanine carboxypeptidase	3.84	3.77	3.21	CpxAR [18]
*dacD*	D-alanyl-D-alanine carboxypeptidase	3.78	3.71	3.45	
*emtA*	Murein transglycosylase E	2.04	1.52	1.41	
*mltA*	Murein transglycosylase A	2.18	2.15	2.12	
*mltD*	Murein transglycosylase D	n.s.	1.53	2.10	
*yfhD*	Lytic transglycosylase F	n.s.	1.61	2.10	
**PROTON-MOTIVE FORCE DISSIPATION**					
*pspA*	Phage shock protein A	4.08	2.84	2.42	PspF [1, 7]
*pspD*	Phage shock protein D	2.88	1.86	n.s.	PspF [1, 7]
*pspE*	Thiosulfate sulfurtransferase PspE	2.36	1.62	n.s.	PspF [1, 7]
*pspG*	Phage shock protein G	4.08	2.75	2.38	PspF [1, 7, 12]

*Highlighted fold changes correspond to up-regulated (dark gray) and down-regulated (light gray) genes. (n.s.), fold changes with p > 0.05*.

*Jovanovic and Model, 1997 [1]; Danese and Silhavy, 1998[2]; Raivio et al., 2000 [3]; De Wulf et al., 2002 [4]; Oshima et al., 2002 [5]; Batchelor et al., 2005 [6]; Darwin, 2005 [7]; Hirakawa et al., 2005 [8]; Dorel et al., 2006 [9]; Yoshida et al., 2006 [10]; Yamamoto et al., 2008 [11]; Bury-Moné et al., 2009 [12]; Price and Raivio, 2009 [13]; Appia-Ayme et al., 2011 [14]; Hu et al., 2011 [15]; Weatherspoon-Griffin et al., 2011 [16]; Perkins et al., 2013 [17]; Raivio et al., 2013 [18]; Rosner and Martin, 2013 [19]*.

The *tolABQR* and *ybgC* genes were also induced, but to lesser extents (2.15- to 3.80-fold at 45 min). These genes encode components of the YbgC-YbgF-TolQ-R-A-B-Pal Cell Envelope Complex, also known as the Tol-Pal system (although *ybgF* and *pal* were not significantly induced). The genes of the Tol-Pal system are, like *degP* and *spy*, CpxAR induced (Bury-Moné et al., 2009). The Tol-Pal system has a role in the maintenance of cell-envelope integrity (Lazzaroni et al., 1999; Cascales et al., 2000) and mutants (*tolA* or *pal*) exhibit an extra-cytoplasmic stress response characterized by a dramatic increase in the transcription of *htrA* (Vines et al., 2005). Two other CpxAR-regulated genes (*yncJ* and *yjfN*), encoding envelope proteins, were also induced in EWMM at 45°C (Table 5) and these may play roles associated with membrane integrity (Raivio et al., 2013).

An induction of genes involved in cell permeability was also apparent with several porin-encoding genes exhibiting differential expression in response to EWMM exposure: *ompC, ompX* and *ompS* were up-regulated (2.53-, 2.19-, and 2.11-fold, respectively, at 45 min; Table 5); whereas *ompF* and *nmpC* (named also *ompD*, the major porin of *Salmonella*) were down-regulated (0.33- and 0.32-fold, respectively, at 45 min; Table 5). Porins enable the diffusion of solutes through the OM and their regulation is controlled through their association with distinct sets of regulons (e.g., CpxAR, RpoE/σ^E^, and OmpR/EnvZ) in response to a wide variety of environmental conditions such as pH, osmolarity, temperature, toxins and growth phase (Pratt and Silhavy, 1996; Table 5). The expression effects observed here are consistent with CpxAR control either by direct CpxAR-mediated up- and down-regulation of *ompC* and *ompF*, respectively, or by indirect control through other transcription factors such as OmpR/EnvZ (Batchelor et al., 2005; Dorel et al., 2006; Lin et al., 2012).

The CpxAR- and BaeSR-controlled *acrD* gene was induced (3.60-fold at 45 min) in EWMM (Table 5). This gene specifies a multidrug-efflux system that removes antimicrobial compounds (e.g., aminoglycosides) from the bacterial cell. In contrast, the *mdtABC* operon specifying another drug-efflux pump that is also controlled by CpxAR and BaeSR, was not affected. This observation is consistent with previous studies showing increased expression of *acrD*, but not *mdtABCD*, under conditions that activated CpxAR but not BaeSR (Rosenberg et al., 2000; Price and Raivio, 2009). This suggests that the *acrD* induction seen here is CpxAR, not BaeSR, dependent. The *emrD* gene also encodes a multidrug efflux pump that was induced in EWMM (3.34-fold at 45 min, Table 5). This gene may be involved in adaptation to energy-shock induced by exposure to uncouplers of oxidative phosphorylation (Naroditskaya et al., 1993).

Seven genes encoding peptidoglycan hydrolases involved in metabolism and turn-over of cell-wall peptidoglycan were also induced. These include the *dacC* and *dacD*, encoding the DD-carboxypeptidases PBP6 and PBP6b, and *amiC* encoding a MurNAc-L-Ala amidase (Table 5). The *mltA, mltD, emtA*, and *yfhD* genes encoding lytic endotransglycosylases were also induced. The *amiC* and *dacC* genes are positively regulated by CpxAR and it is suggested that their up-regulation induces a remodeling of the peptidoglycan in response to environmental challenge (Weatherspoon-Griffin et al., 2011; Raivio et al., 2013).

The pspADEG genes were also induced in EWMM with the strongest induction observed at 7 min and weakest at 45 min (Table 5). These are “phage-shock protein” genes (*pspABCDEFG*) that are induced in response to various membrane-altering stresses and are required for survival at high pH (in *E. coli*; Weiner and Model, 1994). It is suggested that dissipation of the proton-motive force (pmf) acts as the inducing signal for *psp* expression (Darwin, 2005; Jovanovic et al., 2006).

In summary, the above indicate a major induction of membrane-stress related genes in cells exposed to EWMM at 45°C. This effect is consistent with significant CpxAR- and PspF-dependent gene activation.

#### Induction of genes related to the heat-shock response

Incubation of *S*. Enteritidis in EWMM at 45°C caused up regulation of genes (*groEL, groES, grpE*, SEN1800 and *htpG*) encoding heat-shock proteins (3.32- to 16.68-fold induced at 45 min, Table 6). The time-dependent expression data indicate that maximum expression was achieved rapidly (at 7–25 min), following which expression declined (at 45 min). This pattern is distinct from that seen for most of the other groups of differentially regulated genes highlighted here, but has been observed previously, in *E. coli*, in response to sudden temperature up-shift (30–42°C) (Arsène et al., 2000; Guisbert et al., 2008) where it is considered to reflect the need for a rapid adaptation to temperature up-shift. Heat-shock expression is largely controlled by the alternative sigma factor, RpoH, and provides protection against heat by stabilizing stress-denatured proteins (Rouvière et al., 1995).

**Table 6:** **Temperature-related genes**.

**Gene**	**Function**	**Fold change**		
		**7 min**	**25 min**	**45 min**
*htpG*	Heat shock protein 90	4.08	3.85	3.57
*groEL*	Heat shock protein; chaperonin GroEL	8.18	9.93	8.94
*groES*	Heat shock protein; co-chaperonin GroES	5.86	6.66	5.74
*grpE*	Heat shock protein GrpE; nucleotide exchange factor for DnaKJ chaperone	5.36	3.69	3.32
SEN1800	Heat shock protein; HSP20-like chaperone	14.43	22.26	16.68

*Highlighted fold changes correspond to up-regulated (dark gray) and down-regulated (light gray) genes. (n.s.), fold changes with p > 0.05*.

#### Induction of genes involved in a translation stress response

The *relBE* genes, encoding the RelE cytotoxin and RelB antitoxin, were induced in EWMM (3.47- and 3.26-fold for *relE* and *relB*, respectively, at 45 min). Overexpression of RelE is known to inhibit translation and cell growth (Gerdes et al., 2005). The RelBE system is thought to act as a ppGpp-independent stress-response regulator that blocks translation during amino-acid starvation or nutritional stress (Christensen et al., 2001). In addition, four genes encoding ribosome-binding proteins that also shutdown translation (Starosta et al., 2014) under conditions of stress (stationary-phase, cold shock or low energy status) were likewise induced: RaiA (pY, YfiA), the ribosome-associated inhibitor A (*raiA*, 1.91 fold at 45 min); RMF, the ribosome-modulation factor (*rmf*, 5.76- fold at 45 min); EttA (YjjK), the “energy-dependent translational throttle A” (*ettA*, 2.13- fold at 45 min); and SRA (RpsV, S22), the stationary-phase-induced ribosome-associated protein (*rspV*, 4.63-fold at 45 min). These observations suggest that EWMM exposure induces a translational shutdown.

#### Repression of genes involved in amino acid biosynthesis and uptake

Genes involved in the synthesis and transport of amino acids were generally repressed by exposure of *S*. Enteritidis to EWMM at 45°C (Table 7). The amino acids affected include the branched chain group (Val, Leu and Ile), aromatic amino acids (tryptophan, phenylalanine, and tyrosine), threonine, arginine, cysteine, and asparagine.

**Table 7:** **Metabolism and transport of amino acids**.

**Gene**	**Function**	**Fold Change**		
		**7 min**	**25 min**	**45 min**
**BRANCHED CHAIN AMINO ACIDS**				
*ilvC*	Ketol-acid reductoisomerase	0.14	0.09	0.06
*ilvD*	Dihydroxy-acid dehydratase	n.s.	0.70	0.46
*ilvE*	Branched-chain amino acid aminotransferase	0.42	0.45	0.36
*ilvG*	Acetolactate synthase 2 catalytic subunit	0.50	0.49	0.41
*ilvM*	Acetolactate synthase 2 regulatory subunit	0.50	0.49	0.37
*leuA*	2-isopropylmalate synthase	n.s.	0.38	0.16
*leuB*	3-isopropylmalate dehydrogenase	n.s.	0.38	0.17
*leuC*	Isopropylmalate isomerase large subunit	n.s.	0.45	0.18
*leuD*	Isopropylmalate isomerase small subunit	n.s.	0.61	0.33
*livF*	Leucine/isoleucine/valine transporter ATP-binding subunit	n.s.	0.67	0.48
*livG*	Leucine/isoleucine/valine transporter ATP-binding subunit	n.s.	0.46	0.27
*livK*	Leucine-specific binding protein	n.s.	0.39	0.22
*livM*	Leucine/isoleucine/valine transporter permease subunit	n.s.	0.51	0.29
*yeaS*	Leucine export protein LeuE	0.54	0.45	0.32
**AROMATIC AMINO ACIDS**				
*aroD*	3-dehydroquinate dehydratase	n.s.	n.s.	0.48
*aroP*	Aromatic amino acid transporter	n.s.	0.55	0.35
*aroG*	Phospho-2-dehydro-3-deoxyheptonate aldolase	n.s.	0.35	0.22
*mtr*	Tryptophan permease	n.s.	0.37	0.26
*pheA*	Bifunctional chorismate mutase/prephenate dehydratase	0.27	0.35	0.36
*trpA*	Tryptophan synthase subunit alpha	n.s.	0.61	0.34
*trpB*	Tryptophan synthase subunit beta	n.s.	0.53	0.29
*trpC*	Bifunctional indole-3-glycerol phosphate synthase/phosphoribosylanthranilate isomerase	0.43	0.34	0.19
*trpD*	Bifunctional glutamine amidotransferase/anthranilate phosphoribosyltransferase	n.s.	0.34	0.21
*trpE*	Anthranilate synthase component I	n.s.	0.34	0.15
**THREONINE**				
*thrA*	Bifunctional aspartokinase I/homeserine dehydrogenase I	0.29	0.28	0.15
*thrB*	Homoserine kinase	0.33	0.38	0.22
*thrC*	Threonine synthase	n.s.	n.s.	0.38
*thrS*	Threonyl-tRNA synthetase	0.16	0.19	0.20
**ARGININE**				
*adi*	Arginine decarboxylase	0.54	0.30	0.24
*artM*	Arginine transporter permease subunit ArtM	0.51	0.42	0.42
*yjdE*	Arginine:agmatin antiporter	0.49	0.30	0.23
**CYSTEINE**				
*cysA*	Sulfate/thiosulfate transporter subunit	0.36	0.34	0.22
*cysB*	Transcriptional regulator CysB	n.s.	0.38	0.21
*cysC*	Adenylylsulfate kinase	n.s.	0.61	0.3
*cysD*	Sulfate adenylyltransferase subunit 2	n.s.	n.s.	0.41
*cysH*	Phosphoadenosine phosphosulfate reductase	n.s.	0.63	0.46
*cysI*	Sulfite reductase subunit beta	n.s.	0.76	0.43
*cysJ*	Sulfite reductase (NADPH) flavoprotein beta subunit	n.s.	n.s.	0.42
*cysM*	Cysteine synthase B	0.44	0.49	0.49
*cysN*	Sulfate adenylyltransferase subunit 1	n.s.	n.s.	0.43
*cysP*	Thiosulfate transporter subunit	n.s.	0.63	0.38
*cysU*	Sulfate/thiosulfate transporter subunit	n.s.	0.45	0.26
*cysW*	Sulfate/thiosulfate transporter permease subunit	0.31	0.37	0.24
*yedO*	D-cysteine desulfhydrase	0.42	0.42	0.40
**ASPARAGINE, GLUTAMATE**				
*asnA*	Asparagine synthetase AsnA	n.s.	n.s.	0.25
*asnB*	Asparagine synthetase B	0.36	0.37	0.36
*gltB*	Glutamate synthase subunit alpha	0.37	0.49	0.30
*gltD*	Glutamate synthase subunit beta	n.s.	0.68	0.41
*gltJ*	Glutamate/aspartate transport system permease protein GltJ	0.52	n.s.	0.45
*gltK*	Glutamate/aspartate transport system permease protein GltK	0.48	0.76	0.43
*gltP*	Glutamate/aspartate:proton symporter	0.55	0.54	0.40
**LYSINE**				
*cadB*	Lysine/cadaverine antiporter	0.47	0.49	0.37
*lysA*	Diaminopimelate decarboxylase	0.23	0.11	0.10
*lysC*	Aspartate kinase III	2.77	4.14	5.99
**OTHER AMINO-ACID OR PEPTIDE TRANSPORTERS**				
*dppA*	Periplasmic dipeptide transport protein precursor	n.s.	0.35	0.19
*dppB*	Dipeptide transporter permease DppB	0.37	0.40	0.24
*dppC*	Dipeptide transporter	n.s.	n.s.	0.33
*dppD*	Dipeptide transporter ATP-binding subunit	0.40	0.51	0.30
*dppF*	Dipeptide transporter ATP-binding subunit	0.44	0.55	0.39
*oppA*	Periplasmic oligopeptide-binding protein precursor (OppA)	n.s.	0.64	0.36
*oppB*	Oligopeptide transporter permease	n.s.	0.54	0.27
*oppC*	Oligopeptide transport system permease protein (OppC)	0.47	0.49	0.34
*oppD*	Oligopeptide transporter ATP-binding component	0.57	0.74	0.46
*yecC*	Putative amino-acid ABC transporter ATP-binding protein	0.41	0.41	0.40
*yecS*	Putative ABC transporter membrane protein	0.38	0.40	0.41

*Highlighted fold changes correspond to up-regulated (dark gray) and down-regulated (light gray) genes. (n.s.), fold changes with p > 0.05*.

In contrast to the general trend, *lysC* was up-regulated (5.99-fold change at 45 min). Consistent with this induction, *lysC* is reported to be required for viability in egg white at 37°C (Clavijo et al., 2006). *lysC* encodes aspartate kinase involved in lysine, threonine and methionine biosynthesis and in the initial step of diaminopimelate (DAP) synthesis, required for peptidoglycan production (Rodionov, 2003). However, *lysA*, required for the final step of lysine synthesis, was down-regulated (0.10-fold change at 45 min). It is possible that the increased expression of *lysC* reflects the need to regenerate peptidoglycan, which may also explain the slight induction of the *dapABDE* genes (n.s. to 1.81-fold at 45 min data not shown).

The *oppABC* operon, encoding an oligopeptide permease, and *dppABCDF*, specifying a dipeptide permease, were both down-regulated in EWMM (Table 7). These ABC transporters constitute a major route for peptide uptake (Hosie and Poole, 2001; Davidson and Chen, 2004). Their expression is expected to be dependent upon the availability of amino acids and need for protein synthesis (Sharma et al., 2007).

#### Repression of genes involved in motility and chemotaxis

Twenty eight genes involved in motility and chemotaxis were down-regulated (0.05- to 0.42-fold at 45 min, Table 8) with the degree of repression increasing over time. A strong repression was seen for the *flhCD* operon, the class I master operon that encodes the FlhCD regulatory complex. This complex is the principal regulator of bacterial flagellum biogenesis and swarming migration (Claret and Hughes, 2002). The class II FlhDC_−_regulated genes, which encode the flagella basal body export machinery, were also down-regulated, in particular *flgKL, fliDST, flgMN*, and *fliAZ*. The *fliA* gene specifies the flagellum-specific sigma factor, σ^28^, that regulates the class III genes (Hughes et al., 1993). The class III genes (*motAB, cheAW, cheRBYZ, cheM*, SEN3058, *tcp, tsr*, and *fliB*), encoding chemotaxis proteins and structural subunits of the flagellum, were also down-regulated. The FlhDC-regulated *ycgR* and *yhjH* genes (Ko and Park, 2000) were also strongly repressed (0.08- and 0.18-fold at 45 min respectively; Table 8). The *ycgR* gene regulates flagellar motility and *yhjH* is involved in regulation of the switch from flagellar motility to sessile behavior and curli expression.

**Table 8:** **Motility and taxis**.

**Gene**	**Function**	**Fold change**		
		**7 min**	**25 min**	**45 min**
**FLAGELLA BIOSYNTHESIS**				
*flgK*	Flagellar hook-associated protein FlgK	n.s.	0.15	0.10
*flgL*	Flagellar hook-associated protein FlgL	n.s.	0.28	0.18
*flgM*	Anti-sigma28 factor FlgM	n.s.	0.28	0.14
*flgN*	Flagella synthesis chaperone protein FlgN	n.s.	0.26	0.15
*flhB*	Flagellar biosynthesis protein FlhB	0.26	0.25	0.23
*flhC*	Transcriptional activator FlhC	0.61	0.52	0.41
*flhD*	Transcriptional activator FlhD	0.51	0.40	0.34
*fliA*	Flagellar biosynthesis sigma factor	n.s.	0.12	0.07
*fliB*	Lysine-N-methylase	0.14	0.08	0.06
*fliD*	Flagellar capping protein	n.s.	0.18	0.07
*fliS*	Flagellar protein FliS	n.s.	0.18	0.07
*fliT*	Flagellar biosynthesis protein FliT	n.s.	0.16	0.06
*fliZ*	Flagella biosynthesis protein FliZ	n.s.	0.14	0.08
*motA*	Flagellar motor protein MotA	0.37	0.20	0.14
*motB*	Flagellar motor protein MotB	n.s.	0.24	0.14
**CHEMOTAXIS**				
*cheA*	Chemotaxis protein CheA	n.s.	0.25	0.14
*cheB*	Chemotaxis-specific methylesterase	0.39	0.32	0.25
*cheM*	Methyl-accepting chemotaxis protein II	n.s.	0.20	0.12
*cheR*	Chemotaxis methyltransferase CheR	0.33	0.22	0.16
*cheW*	Purine-binding chemotaxis protein	n.s.	0.40	0.21
*cheY*	Chemotaxis regulatory protein CheY	n.s.	0.42	0.24
*cheZ*	Chemotaxis regulator CheZ	n.s.	0.42	0.27
SEN2296	Chemotaxis protein CheV	0.18	0.10	0.06
SEN3058	Methyl-accepting chemotaxis protein II	n.s.	0.19	0.10
*tcp*	Methyl-accepting chemotaxis citrate transducer	0.15	0.08	0.05
*tsr*	Methyl-accepting chemotaxis protein	n.s.	0.21	0.14
**MOTILITY REGULATION**				
*aer*	Aerotaxis receptor protein	n.s.	0.50	0.42
*lrhA*	Transcriptional regulator	0.37	0.29	0.20
*ycgR*	Flagellar brake protein YcgR	n.s.	0.20	0.08
*yhjH*	Cyclic-guanylate-specific phosphodiesterase	n.s.	0.23	0.18

*Highlighted fold changes correspond to up-regulated (dark gray) and down-regulated (light gray) genes. (n.s.): fold changes with p > 0.05*.

The *lrhA* gene, encoding “LysR homolog A,” was 0.20-fold down-regulated at 45 min. LrhA regulates the transcription of genes involved in the synthesis of type 1 fimbriae (Blumer, 2005). Indirectly, this protein also regulates the transcription of several genes involved in motility, chemotaxis and flagellum synthesis by directly controlling the expression of the master regulator FlhDC (Lehnen et al., 2002). The expression of flagella/motility/chemotaxis components is highly regulated by multiple environmental stimuli including stress factors such as heat-shock (Walker et al., 1999), extreme pH (Maurer et al., 2005), envelope stress (CpxAR dependent, De Wulf et al., 2002) and low iron content (via RyhB in *Salmonella* Typhimurum; Kim and Kwon, 2013b). These stresses are relevant to egg white exposure and so may well explain the down shift in motility/taxis genes observed here.

#### Induction of the Kdp potassium uptake system

Egg white exposure resulted in a significant stimulation of the genes specifying the high-affinity K^+^ uptake system, Kdp. Kdp is a P-type ATPase composed of the K^+^ transporter, KdpABC, and the two-component regulator, KdpDE. *kdpABC* and *kdpD* were induced by EWMM at 45°C (3.28- to 7.60-fold at 45 min, Table 9), although *kdpE* was not affected. Potassium is a major cytoplasmic cation in bacteria being involved in maintenance of osmotic pressure and in regulation of cytoplasmic pH. The Kdp system is activated when K^+^ is limited, when cytoplasmic pH is suboptimal or when turgor pressure is decreased (Epstein, 2003). A variety of environmental conditions including the pH, growth temperature and the concentration of other cationic solutes are known to additionally modulate the strength of the stimulus perceived by KdpD (Asha and Gowrishankar, 1993).

**Table 9:** **High-affinity potassium uptake**.

**Gene**	**Function**	**Fold change**		
		**7 min**	**25 min**	**45 min**
*kdpA*	Potassium-transporting ATPase subunit A	2.75	3.60	5.74
*kdpB*	Potassium-transporting ATPase subunit B	2.68	3.16	5.28
*kdpC*	Potassium-transporting ATPase subunit C	3.17	5.05	.60
*kdpD*	Sensor kinase KdpD	n.s.	2.29	3.28

*Highlighted fold changes correspond to up-regulated (dark gray) and down-regulated (light gray) genes. (n.s.), fold changes with p > 0.05*.

### Shift in energy metabolism and catabolism

#### Repression of genes involved in respiration

Many of the genes encoding proteins involved in energy generation under aerobic and/or anaerobic conditions were down regulated upon exposure to the EWMM at 45°C (0.05- to 0.70-fold at 45 min, Table 10). These include genes encoding for cytochrome *bo* (*cyoABCDE*), succinate dehydrogenase (*sdhCDAB)*, NADH dehydrogenases I and II (*ndh, nuoABCDEFGHIJKLM*), formate dehydrogenase-O (*fdoIGH*), anaerobic glycerol3-phosphate dehydrogenase (*glpABC*), formate dehydrogenase-H (*fdhF*), hydrogenases 1, 2, and 3 (*hyaD, hybADEF, hycCDEFGHI, hydN, hypBC*,), pyruvate formate lyase-activating enzyme (*pflABEF, yfiD*), and the reductases of nitrite, nitrate, sulfite, dimethylsulphoxide and fumarate (*nirBD, napADF, asrAC dmsABC, torT*, and *frdABD*, respectively). This set of repressed genes includes many that specify iron-containing proteins (Table 10). Indeed, many of the genes listed in Table 10 are reported to be RyhB/RfrAB, and/or Fur controlled in the Enterobacteriaceae (Table 10).

**Table 10:** **Energy metabolism**.

**Gene**	**Function**	**Fold change**			**Fe content**	**Fur/RyhB regulation**	**Other regulation**
		**7 min**	**25 min**	**45 min**			
*asrA*	Anaerobic sulfite reductase subunit A	0.39	0.36	0.26	Yes		
*asrC*	Anaerobic sulfite reductase subunit C	0.56	0.47	0.42	Yes		
*cyoA*	Cytochrome o ubiquinol oxidase subunit II	n.s.	0.71	0.39	No	[3, 5, 10, 16]	CpxAR [13]
*cyoB*	Cytochrome o ubiquinol oxidase subunit I	n.s.	n.s.	0.41	Yes	[3, 5, 10, 16]	FlhDC [4], CpxAR[13]
*cyoC*	Cytochrome o ubiquinol oxidase subunit III	0.52	0.64	0.36	No	[3, 5, 10, 16]	FlhDC [4], CpxAR[13] PspF [7]
*cyoD*	Cytochrome o ubiquinol oxidase subunit IV	0.59	0.79	0.46	No	[3, 5, 10, 16]	FlhDC [4], CpxAR[13]
*dmsA*	Anaerobic dimethyl sulfoxide reductase chain A precursor	0.35	0.30	0.29	Yes	[10]	FlhDC[1, 4, 15], PspF [7]
*dmsB*	Anaerobic dimethyl sulfoxide reductase chain B	0.40	0.33	0.29	Yes	[10]	FlhDC [1, 4], PspF [7]
*dmsC*	Anaerobic dimethyl sulfoxide reductase chain C	0.43	0.42	0.39	No	[3, 10]	FlhD [1]
*fdhE*	Formate dehydrogenase accessory protein	0.38	0.42	0.43	Yes		
*fdhF*	Formate dehydrogenase H	0.39	0.35	0.27	Yes		
*fdoG*	Formate dehydrogenase-O, major subunit	0.15	0.23	0.16	Yes	[5]	
*fdoH*	Formate dehydrogenase-O beta subunit	0.16	0.23	0.20	Yes	[5]	
*fdoI*	Formate dehydrogenase-O subunit gamma	0.11	0.17	0.13	Yes	[5]	PspF [7]
*frdA*	Fumarate reductase flavoprotein subunit	0.58	0.44	0.38	No	[3, 5, 10, 11, 12, 14]	FlhDC [4]
*frdB*	Fumarate reductase iron-sulfur subunit	0.59	0.46	0.40	Yes	[3, 5, 10]	FlhDC [4]
*frdD*	Fumarate reductase subunit D	n.s.	0.50	0.48	No	[3, 5, 10]	FlhDC [4]
*glpA*	Sn-glycerol-3-phosphate dehydrogenase subunit A	0.50	0.33	0.25	No	[3, 10]	FlhDC [1, 4], PspF [8], CpxAR [8]
*glpB*	Anaerobic glycerol-3-phosphate dehydrogenase subunit B	n.s.	0.50	0.43	No	[3, 10]	FlhDC [1, 4], PspF [8], CpxAR [8]
*glpC*	Sn-glycerol-3-phosphate dehydrogenase subunit C	0.47	0.35	0.29	Yes	[3, 10]	FlhDC [1], PspF [8], CpxAR [8]
*hyaD*	Hydrogenase 1 maturation protease	0.55	0.49	0.47	No		
*hybA*	Hydrogenase 2 protein HybA	0.30	0.31	0.30	Yes	[5]	FlhDC [4]
*hybD*	Hydrogenase 2 maturation endopeptidase	0.48	0.58	0.70	No	[5]	FlhDC [4]
*hybE*	Hydrogenase 2-specific chaperone	0.42	n.s.	n.s.		[5]	
*hybF*	Hydrogenase nickel incorporation protein HybF	0.34	0.47	0.56	No	[5]	FlhDC [4]
*hycA*	Formate hydrogenlyase regulatory protein HycA	n.s.	0.40	0.36	No		
*hycC*	Formate hydrogenlyase subunit 3	n.s.	0.51	0.38	No		
*hycD*	Formate hydrogenlyase subunit 4	n.s.	0.42	0.27	No		
*hycE*	Formate hydrogenlyase subunit 5	0.47	0.33	0.29	Yes		
*hycF*	Formate hydrogenlyase complex iron-sulfur subunit	0.40	0.28	0.20	Yes		PspF [7]
*hycG*	Formate hydrogenlyase subunit 7	0.54	0.44	0.45	Yes		PspF [7]
*hycH*	Formate hydrogenlyase maturation protein	0.34	0.25	0.21	No		
*hycI*	Hydrogenase 3 maturation protease	0.35	0.24	0.21	No		PspF [7]
*hydN*	Electron transport protein HydN	0.21	0.22	0.18	Yes		
*hypB*	Hydrogenase nickel incorporation protein HypB	n.s.	0.56	0.49	No		
*hypC*	Hydrogenase assembly chaperone	0.60	0.50	0.47	Yes		
*napA*	Nitrate reductase catalytic subunit	n.s.	0.38	0.35	Yes	[3]	FlhDC [4]
*napD*	Assembly protein for periplasmic nitrate reductase	n.s.	0.14	0.10	No	[3]	FlhDC [4]
*napF*	Ferredoxin	0.15	0.11	0.07	Yes	[3]	FlhDC [4]
*ndh*	NADH dehydrogenase	0.34	0.32	0.31	No		
*nirB*	Nitrite reductase large subunit	n.s.	0.40	0.37	Yes	[3, 14, 16]	PspF [7]
*nirD*	Nitrite reductase small subunit	n.s.	0.46	0.48		[3, 16]	
*nuoA*	NADH dehydrogenase subunit A	0.38	0.34	0.33	No	[3, 5]	CpxAR [13]
*nuoB*	NADH dehydrogenase subunit B	0.41	0.36	0.35	Yes	[3, 5]	CpxAR [13]
*nuoC*	Bifunctional NADH:ubiquinone oxidoreductase subunit C/D	0.30	0.35	0.33	No	[3, 5]	CpxAR [13]
*nuoE*	NADH dehydrogenase subunit E	0.34	0.40	0.37	Yes	[3, 5]	CpxAR [13]
*nuoF*	NADH dehydrogenase I subunit F	0.35	0.44	0.40	Yes	[3, 5]	CpxAR [13]
*nuoG*	NADH dehydrogenase subunit G	0.38	0.50	0.52	Yes	[3, 5]	CpxAR [13]
*nuoH*	NADH dehydrogenase subunit H	0.22	0.34	0.33	No	[3, 5]	CpxAR [13]
*nuoI*	NADH dehydrogenase subunit I	0.24	0.36	0.36	Yes	[3, 5]	CpxAR [13]
*nuoJ*	NADH dehydrogenase subunit J	0.27	0.39	0.39	No	[3, 5]	CpxAR [13]
*nuoK*	NADH dehydrogenase subunit K	0.27	0.42	0.41	No	[3, 5]	CpxAR [13]
*nuoL*	NADH dehydrogenase subunit L	0.31	0.56	0.56	No	[3, 5]	CpxAR [13]
*nuoM*	NADH dehydrogenase subunit M	0.39	0.61	0.64	No	[3, 5]	CpxAR [13]
*pflA*	Pyruvate formate lyase-activating enzyme 1	0.65	0.56	0.44	Yes		
*pflB*	Formate acetyltransferase 1	n.s.	0.45	0.50	No		
*pflE*	Putative pyruvate formate-lyase 3 activating enzyme	0.14	0.15	0.12	No		
*pflF*	Putative formate acetyltransferase 3	0.14	0.16	0.14	No	[12]	
*sdhA*	Succinate dehydrogenase flavoprotein subunit	n.s.	0.60	0.33	No	[2, 5, 10, 14]	FlhDC [4], CpxAR[13]
*sdhB*	Succinate dehydrogenase iron-sulfur subunit	n.s.	n.s.	0.45	Yes	[2, 5, 6]	FlhDC [4], CpxAR[13]
*sdhC*	Succinate dehydrogenase cytochrome b556 large membrane subunit	0.50	0.63	0.44	Yes	[2, 5, 6, 9, 14]	FlhDC [4], CpxAR[13]
*sdhD*	Succinate dehydrogenase cytochrome b556 small membrane subunit	0.41	0.60	0.35	Yes	[2, 5, 6, 14]	FlhDC [4], CpxAR[13]
*torT*	TMAO reductase system periplasmic protein TorT	0.45	0.45	0.35	No		
*yfiD*	Autonomous glycyl radical cofactor GrcA, stress-induced alternate pyruvate formate-lyase subunit	0.13	0.05	0.05	No	[9]	FlhDC [4]

*Highlighted fold changes correspond to up-regulated (dark gray) and down-regulated (light gray) genes. (n.s.), fold changes with p > 0.05*.

*Prüß et al., 2001 [1]; Massé and Gottesman, 2002 [2]; McHugh et al., 2003 [3]; Prüß et al., 2003 [4]; Massé et al., 2005 [5]; Zhang et al., 2005 [6]; Jovanovic et al., 2006 [7]; Bury-Moné et al., 2009 [8]; Kumar and Shimizu, 2011 [9]; Troxell et al., 2011 [10]; Kim and Kwon, 2013a [11]; Kim and Kwon, 2013b [12]; Raivio et al., 2013 [13]; Wright et al., 2013 [14]; Yang et al., 2013 [15]; Calderón et al., 2014 [16]*.

The *E. coli nuo, ndh* and *sdh* genes have also been reported to be repressed at high pH (Maurer et al., 2005) possibly through control by CpxAR (note that the *cyo* genes are also CpxAR repressed Raivio et al., 2013). The *fdo* and *hyc* genes are also regulated by PspF in response to high pH response (Jovanovic et al., 2006) and other genes are regulated by FlhDC (Table 10).

In summary, we observed down-regulation of most of the genes involved in energy generation by respiration and these effects are likely controlled by Fur/RfrAB, CpxAR, PspF, and/or FlhDC.

#### Induction of genes involved in utilization of hexonates and hexuronates and carbohydrate metabolism

Surprisingly, exposure to EWMM at 45°C strongly induced three distinct gene clusters involved in hexonate/hexuronate utilization: the *dgoRKADT* operon; the *uxuAB-uxaC* operon; and the SEN1433-6 genes. The *dgo* genes were 13.59- to 31.13-fold induced (at 45 min; Table 11) in EWMM. Their general function is believed to be in utilization of D-galactonate and 2-keto-3-deoxygalactonate. *dgoT* is inferred to encode a D-galactonate uptake system; *dgoA, dgoK* and *dgoD* are suggested to code for enzymes involved in the conversion of D-galactonate to pyruvate and glyceraldehyde-3-phosphate, and *dgoR* encodes a GntR/FadR-related regulator likely acting as a D-galactonate-responsive transcriptional repressor of the *dgo* operon (Cooper, 1978; Zhou and Rudd, 2013). The *uxuAB-uxaC* operon is believed to be involved in mannonate utilization; these genes were induced by levels (10.68- to 28.2-fold, 45 min, Table 11) similar to those observed for the *dgo* genes. The SEN1433-5 genes form a putative operon adjacent to the functionally-related and divergent SEN1436 gene. They are induced by 5.17- to 33.38-fold (at 45 min, Table 11), similar to *dgo* and *uxuAB-uxaC*. The genes of the SEN1432-6 cluster specify three enzymes (two suspected dehydrogenases and one dehydratase), likely to be involved in hexonate utilization, and a proposed hexonate transporter.

**Table 11:** **Hexonate and hexuronate utilization and carbohydrate metabolism**.

**Gene**	**Function**	**Fold change**		
		**7 min**	**25 min**	**45 min**
**GLYCOLYSIS/HEXOSE METABOLISM**				
*fbaB*	Fructose-bisphosphate aldolase	2.27	2.98	4.28
*frwB*	Putative fructose-like phosphotransferase EIIB subunit 2	2.28	2.52	2.56
*frwC*	Putative fructose-like permease EIIC subunit 2	1.59	2.35	2.17
*gapA*	Glyceraldehyde 3-phosphate dehydrogenase A	3.10	2.33	2.02
*gpmA*	Phosphoglycerate mutase 1	1.48	1.94	2.14
*pfkB*	6-phosphofructokinase 2	4.53	5.47	4.87
*ptsA (frwA)*	Phosphoenolpyruvate-protein phosphotransferase	1.69	2.26	2.53
*ptsH*	Phosphohistidinoprotein-hexose phosphotransferase component of PTS system (HPr)	1.76	2.07	2.28
*pykA*	Pyruvate kinase	n.s.	1.73	1.99
*talC*	Fructose-6-phosphate aldolase	2.88	5.38	5.62
*treB*	Trehalose(maltose)-specific PTS system components IIBC	2.52	2.49	2.27
**ONE CARBON PATHWAY**				
*gcvA*	DNA-binding transcriptional activator GcvA	n.s.	0.62	0.39
*gcvH*	Glycine cleavage system protein H	1.94	2.00	2.15
*gcvT*	Glycine cleavage system aminomethyltransferase T	2.13	2.08	2.12
*kbl*	2-amino-3-ketobutyrate coenzyme A ligase	2.65	2.20	2.00
*tdh*	L-threonine 3-dehydrogenase	2.23	1.80	1.64
*glyA*	Serine hydroxymethyltransferase	3.40	2.83	2.56
*sdaA*	L-serine deaminase 1	3.03	3.54	3.34
*serB*	Phosphoserine phosphatase	2.85	2.40	2.75
**TCA CYCLE**				
*aceE*	Pyruvate dehydrogenase subunit E1	0.21	0.36	0.36
*aceF*	Dihydrolipoamide acetyltransferase	0.23	0.44	0.43
*acnA*	Aconitate hydratase	0.45	0.51	0.37
*acnB*	Bifunctional aconitate hydratase 2/2-methylisocitrate dehydratase	0.29	0.35	0.27
*lpdA*	Dihydrolipoamide dehydrogenase	0.30	0.50	0.48
**MIXED FERMENTATION**				
*ackA*	Acetate kinase	n.s.	2.02	2.23
*adhP*	Alcohol dehydrogenase	n.s.	2.22	1.95
**PENTOSE PHOSPHATE SHUNT (NON-OXIDATIVE BRANCH)**				
*rpiA*	Ribose-5-phosphate isomerase A	3.01	2.24	2.15
*tktA*	Transketolase	2.94	2.91	3.17
**D-GALACTONATE UTILIZATION**				
*dgoA*	D-galactonate dehydratase (SEN3644)	5.87	26.22	24.85
*dgoD*	2-dehydro-3-deoxy-6-phosphogalactonate aldolase (SEN3645)	6.67	23.93	22.03
*dgoK*	2-dehydro-3-deoxygalactonokinase	9.47	34.35	31.13
*dgoR*	Galactonate operon transcriptional repressor	8.96	28.70	27.13
*dgoT*	D-galactonate transporter	n.s.	10.77	13.59
*dkgB*	2,5-diketo-D-gluconate reductase B	2.89	2.19	2.85
*yiaE*	Putative 2-hydroxyacid dehydrogenase	2.05	2.04	2.07
**MANNONATE UTILIZATION**				
SEN2978	Mannonate dehydratase (*uxuA*)	4.39	24.16	28.19
SEN2979	D-mannonate oxidoreductase (*uxuB*)	n.s.	16.77	19.11
SEN2980	Glucuronate isomerase; uronate isomerase; uronic isomerase (*uxaC*)	n.s.	8.81	10.68
**HEXONATE UTILIZATION**				
*eda*	Keto-hydroxyglutarate-aldolase/keto-deoxy-phosphogluconate aldolase	2.09	2.01	1.94
SEN1433	L-idonate 5-dehydrogenase IdnD	3.05	3.21	5.17
SEN1434	Hexonate sugar transporter	n.s.	2.70	5.68
SEN1435	Gluconate 5-dehydrogenase	n.s.	3.07	.40
SEN1436	D-galactonate dehydratase family member SEN1436	n.s.	10.30	33.38

*Highlighted fold changes correspond to up-regulated (dark gray) and down-regulated (light gray) genes. (n.s.), fold changes with p > 0.05*.

A subset of genes encoding phosphotransferase components, involved in the uptake of carbohydrate, was also induced (*ptsH, ptsA(frwA), frwBC, treB*) in EWMM, as were several genes specifying glycolysis and pentose phosphate pathway enzymes (*gapA, gpmA, pykA, fbaB, pfkB, talC, rpiA, tktA*), by 1.99- to 5.62-fold at 45 min (Table 11). Six genes involved in the one carbon pathway and degradation of serine, glycine and threonine to pyruvate and ammonia were also induced by EWMM (*gcvT, gcvH, sdaA, glyA, kbl, tdh, serB*; Table 11). Both the *glyA* and *sdaA* genes of *E. coli* were up-regulated by heat shock (Nonaka et al., 2006; Wolfe et al., 2008; Lüders et al., 2009). Indeed, the acetate kinase (*ackA*) and ethanol dehydrogenase (*adhP*) genes encoding mixed-acid fermentation enzymes were induced (Table 11). In contrast, several genes of the TCA cycle (*sdhCDAB*, Table 10; *lpdA* and *acnAB*, Table 11) were 0.36- to 0.48-fold down-regulated in EWMM.

In summary, exposure to EWMM at 45°C provoked changes in carbohydrate metabolism involving activation of the hexonate/hexuronate, pentose phosphate, glycolysis pathways and repression of the TCA cycle.

### Confirmation of microarray data by qRT-PCR

To confirm the validity of the expression effects observed by microarray analysis, qRT-PCR was used to determine the expression changes of six genes following incubation in EWMM for 45 min at 45°C (Table 12). The six genes selected for analysis were from four different, relevant functional groups and included those displaying the highest differential expression effects (*iroB, fes*, and *ftn* for iron acquisition, *bioB* for biotin synthesis, *dgoK* for hexonate metabolism and *spy* for envelope stress). The qRT-PCR data confirm the expression change of the six genes in response to exposure of *S*. Enteritidis to EWMM, although qRT-PCR indicated a greater degree of regulation (3- to 24-fold higher) than the microarray analysis, as previously reported (Franchini, 2006). Thus, the qRT-PCR data support the induction of the iron restriction, hexonate catabolism, biotin biosynthesis and envelope-stress genes in EWMM and therefore provide support for the reliability of the expression effects revealed by microarray analysis (Tables 2–11).

**Table 12:** **Confirmation of selected genes by qRT-PCR analysis**.

**Gene**	**Function**	**Fold change (45°C, 45 min)**	
		RT-PCR^*^	**Microarray**
*bioB*	Biotin synthetase, biotin synthesis	172.7 (± 34.5)	23.4
*dgoK*	2-dehydro-3-deoxygalactonokinase hexonate metabolism	240.2 (± 44.3)	31.1
*fes*	Ferric enterobactin esterase, iron uptake	285.5 (± 87.5)	11.9
*ftnA*	Ferritin, iron storage	0.76 (± 0.04)	0.19
*iroB*	Salmochelin synthesis, iron uptake	14.9 (± 1.4)	4.1
*spy*	Spheroplast formation	64.5 (± 32.7)	22.2

*Highlighted fold changes correspond to up-regulated (dark gray) and down-regulated (light gray) genes*.

* *Calculation of fold change by qRT-PCR was determined from the number of RNA copies after 45 min of incubation divided by the number of RNA copies at T = 0). A total of two RNA extractions were performed using distinct S. Enteritidis cultures grown on different batches of EWMM. Each of the two RNA extracts thus obtained was subject to qRT-PCR, in triplicate, for each gene. Data were normalized using values for three internal control genes (asmA, emrA, orf32). Standard deviations are calculated from two sets of triplicate data are in parentheses*.

## Discussion

### Overview

Here we describe the first global-transcriptional analysis of the response of *S*. Enteritidis to egg white exposure under bactericidal conditions, providing a unique insight into egg white antibacterial activity. In order to enable this study, an “egg white model medium” (EWMM) was employed that lacks the problematic viscosity of egg white. This medium was shown to retain the bacteriostatic and bactericidal activity of egg white at 30 and 45°C, respectively, which supports previous work showing EWMM to be an appropriate mimick of egg white (Baron et al., 1997; Alabdeh et al., 2011). This bactericidal action clearly requires the combined action of temperature with egg white as *S*. Enteritidis is not subject to substantial killing in TSB at 45°C nor in egg white at lower temperature (30°C). Previous work showed only a moderate bactericidal activity for egg white at 42°C (reduction of less than 2 log_10_ CFU/ml to 3.5 log_10_ CFU/ml for *S*. Enteritidis during a 24–96 h incubation; Guan et al., 2006; Kang et al., 2006; Raspoet et al., 2014) whereas a complete bactericidal effect was seen at 45°C (Alabdeh et al., 2011). This observation matches a previous study (Liot and Anza, 1996) showing that bacterial destruction by liquid egg white treatment (as used in the food industry) is optimal at 42–45°C. Thus, incubation at 45°C in EWMM (as employed here) represents ideal conditions for examination of the response of *S*. Enteritidis to the bactericidal activity of egg white. Relatively short incubation times (7–45 min) were used since prolonged EWMM exposure results in total cell death at 24 h.

### Transcriptional response of *S*. enteritidis to EWMM

**Figure 2:**
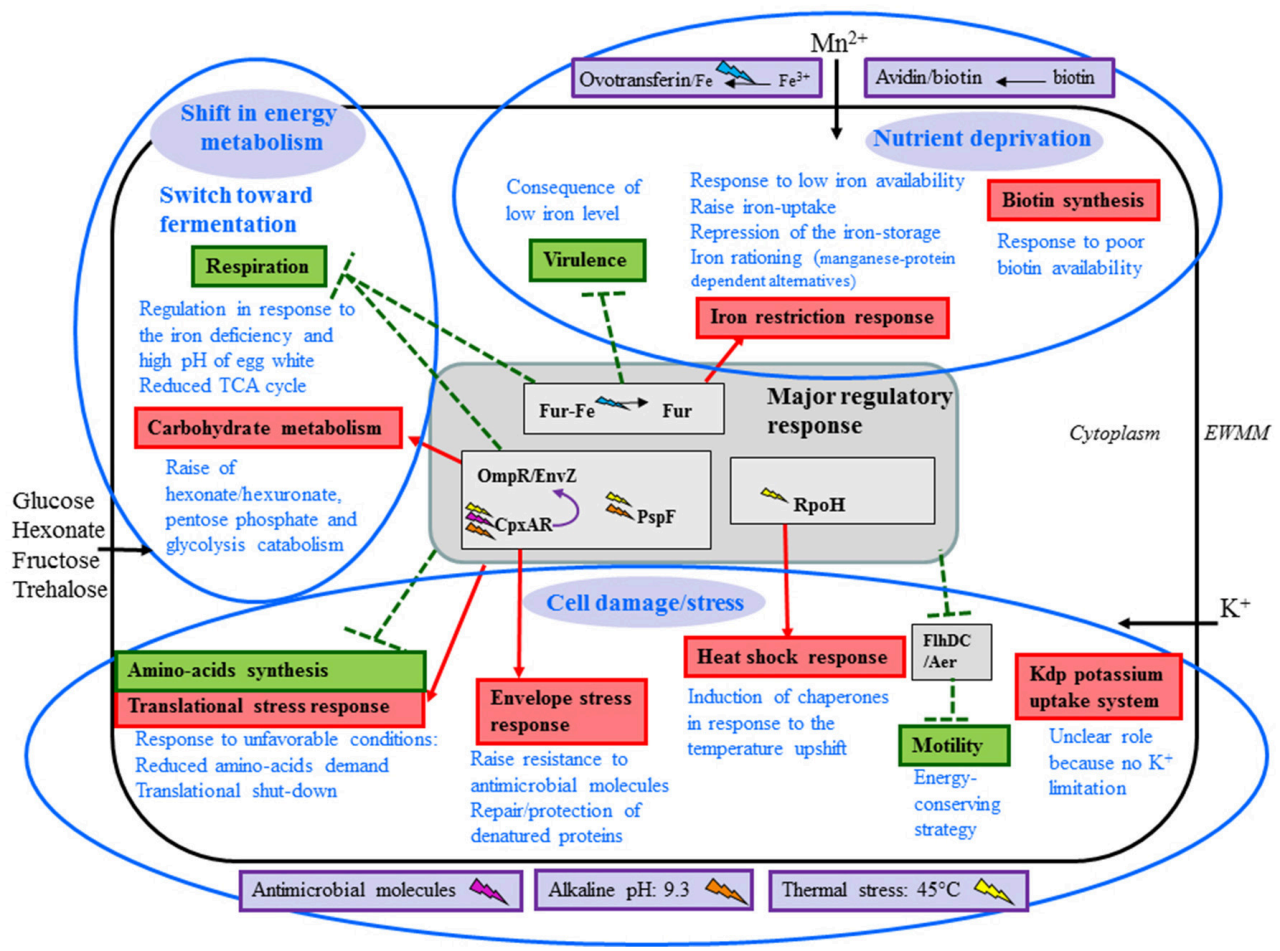
**Summary of physiological response of ***S***. Enteritidis to egg white exposure**. Blue elipses are used to indicate the three major responses elicited by egg white. The central gray box represents the major genetic regulatory responses. Boxes in purple are the egg white factors that are interpreted as having a clear impact on *S*. Enteritidis under the conditions employed here. Systems induced and repressed are indicated in red and green, respectively. Physiological responses are indicated in blue text. Black arrows indicate solute uptake suggested to be triggered by egg white exposure. Red arrows and green broken lines indicate, respectively, activation (red) and repression (green) by corresponding regulators: the origin of the arrow indicates the regulator involved (origins located in the central gray box indicate that all associated regulators are involved). Lighting symbols represent the stimuli that activate regulator responses: pink for antimicrobial molecules, orange for alkaline stress, yellow for thermal stress and blue for low iron conditions.

Incubation of *S*. Enteritidis in EWMM at 45°C provoked a major transcriptional response (18.7% of genes affected at 45 min) indicative of a substantial shift in cellular physiology. Of the differentially-regulated genes, 49.2% were assigned to one of eleven functional classes, as described in the Results section, reflecting specific physiological alterations caused by egg white exposure. Among the remaining genes, most (47.3%) have either unknown or hypothetical function. The eleven functional classes thus defined were divided into three broad categories: nutrient deprivation; cell damage/stress; and a shift in energy metabolism and catabolism (Figure 2). The majority of these changes correlate well with previous findings and with the conditions associated with egg white (Baron et al., 2016).

#### Nutrient deprivation

The strong induction of the *bio* operon (Table 2) is fully consistent with results suggesting a role for the *bioB* gene in egg white survival at 42°C (Raspoet et al., 2014) and can be considered to represent a rational physiological response of *S*. Enteritidis to the poor biotin avaibility in egg white resulting from the presence of avidin, a powerful biotin-chelation protein (Banks et al., 1986; Beckett, 2007) that thus supports the validity of the array data. Likewise, the comprehensive induction of genes involved in the iron-starvation response (Table 3) supports the notion that egg white represents an iron-restricted environment for *Salmonella*. These findings corroborate a number of previous studies suggesting that a major contributor to the bacteria-growth inhibition capacity of egg white is the imposition of iron deficiency, generated largely as a consequence of the strong ferric-iron chelation activity of ovotransferrin (Garibaldi, 1970; Lock and Board, 1992; Baron et al., 1997; Kang et al., 2006). Indeed, previous studies have shown that *S*. Enteritidis iron-acquisition mutants (*entF* and *entF/feoAB*) are attenuated for survival in egg white (Kang et al., 2006) providing additional evidence for the iron-restricted nature of egg white. This effect appears to be coordinated by the global Fe-regulator, Fur, and incorporates the following effects: induction of iron-uptake pathways; down-regulation of iron-storage capacity (along with the probable mobilization of iron stores); induction of the alternative Fe-S cluster manufacturing pathway; iron rationing with replacement of iron-dependent proteins by manganese-dependent alternatives; and induction of manganese uptake to supply Mn^2+^ to the newly-induced Mn-dependent isoenzymes.

The down-regulation of a set of virulence genes (Table 4) associated with the SPI-1 locus (all members of the Fur modulon) correlates with the iron-restriction imposed by egg white (Teixidó et al., 2011).

#### Cell damage/stress

The expression data were consistent with a considerable membrane-stress response (Table 5). The up-regulation of *spy* is consistent with the microscopy observation made on *E. coli* in the same conditions (Jan et al., 2013), where incubation in EWMM for 45 min at 45°C caused formation of spheroplasts. The induction of *degP*, encoding an endoprotease that degrades abnormal proteins, is consistent with previous studies. Mo et al. (2006) showed that DegP is required for survival of *Salmonella* Typhimurium at high temperatures and Raspoet et al. (2014) reveal its role in survival of *S*. Enteritidis in egg white at 42°C. The genes of the Tol-Pal system involved in the maintenance of cell-envelope integrity were also induced.

The observed change in the porin-expression profile (up regulation of *ompC* and down regulation of *ompF*) would be consistent with an attempt by *S*. Enteritidis to protect itself from the antimicrobial activities within egg white. The reciprocal regulation of *ompC* and *ompF* might enable the cell to continue to acquire nutrients whilst limiting exposure to toxins by utilizing OmpC (smaller pore size) in place of OmpF (Nikaido, 1996; Batchelor et al., 2005). Replacing the general-diffusion porins with porins of smaller pore-size is a recognized antibiotic-resistance strategy (Delcour, 2009).

Induction of several peptidoglycan hydrolase genes (*dacC, dacD amiC, mltA, mltD, emtA, yfhD*) was another feature of the microarray data. Up-regulation of *amiC* is already recognized to enhance survival of *E. coli* during treatment with the antimicrobial peptide protamine (Weatherspoon-Griffin et al., 2011). The up-regulation of peptidoglycan hydrolases is suggested to induce a remodeling of the peptidoglycan in response to environmental challenge and so would be fully consistent with an envelope-stress response. However, it should be noted that expression of the major peptidoglycans synthetases (*mcrA, mcrB, pbpA, pbpB, pbpC, ftsI*, and *mtgA*; data not shown) was not significantly affected. Appropriate balance between peptidoglycan synthesis and hydrolysis is critical for cell integrity (Meisel et al., 2003; Kumar et al., 2012). The apparent imbalance in their respective expression levels upon EWMM exposure might result in excessive peptidoglycan hydrolysis leading to loss of integrity of the peptidoglycan layer, which would be expected to promote spheroplast formation as seen for *E. coli* EWMM exposure (Jan et al., 2013).

The induction of the *pspADEG* genes observed here is consistent with the *psp* response previously described in *E. coli* and *Salmonella* Typhimurium where up regulation of *pspA* and *pspG* was triggered by the reduction of the pmf, without effect on other *psp* genes (Lloyd et al., 2004). These results are also consistent with the production of PspA in response to heat stress at 45°C (Hassani et al., 2009). There are at least two factors (ovotransferrin and high pH) in egg white that can cause dissipation of the pmf and which could thus induce the observed *psp* response (Aguilera et al., 2003; Darwin, 2005).

The expression data indicate a major induction of membrane-stress related genes in cells exposed to EWMM at 45°C, likely mediated by the transcriptional regulators CpxR, OmpR and PspF (Figure 2). The probable environmental factors triggering this effect in EWMM are antimicrobial egg white components (Kohanski et al., 2008; Laubacher and Ades, 2008; Farris et al., 2010; Audrain et al., 2013; Evans et al., 2013; Raivio et al., 2013; Raivio, 2014), pmf dissipation (Weiner and Model, 1994; Kleerebezem et al., 1996; Joly et al., 2010) and alkaline pH (Danese and Silhavy, 1998; Thede et al., 2011; Tschauner et al., 2014) along with temperature (Brissette et al., 1990; Danese and Silhavy, 1998; Raivio and Silhavy, 2001; Darwin, 2005; Joly et al., 2010; Raivio, 2014). Previous work has also suggested that maintenance of cell-envelope integrity is an important facet of resistance to egg white (Gantois et al., 2008a), with cell-wall disruption and progressive cell lysis reported as the major mechanisms of egg white-mediated bactericidal action at 45°C for *E. coli* (Jan et al., 2013).

Heat-shock proteins under RpoH control (Rouvière et al., 1995) were also induced (Table 6), presumably in response to the temperature upshift experienced by *S*. Enteritidis upon transfer of the inoculum (37°C) to EWMM (45°C). This type of induction was also observed in *E. coli*, (Arsène et al., 2000; Guisbert et al., 2008) and indicates that *S*. Enteritidis suffers heat-shock under the conditions employed here. In addition, the induction of a “ribosomal-stress response,” and the large-scale repression of genes required for amino-acid biosynthesis and uptake (Table 7), suggest a translational shutdown. It is likely that this translational down-regulation is caused by the unfavorable growth conditions imposed by the hostile conditions of EWMM at 45°C resulting in a block in protein production through interference with ribosome activity possibly arising from reduced amino acid availability or from a direct effect of temperature on the ribosome (Starosta et al., 2014).

It should be noted that amino acid synthesis is considered important for survival in egg white at 37°C (Clavijo et al., 2006; Gantois et al., 2008a). However, the conditions used here (45°C) are bactericidal and so no growth is expected, and thus amino acid production is unlikely to be a major requirement and this would be consistent with a reduced anabolic demand probably resulting from the cessation of growth that occurs upon EWMM exposure.

A further major alteration in expression was seen for motility, as the genes associated with flagella biosynthesis and chemotaxis were subject to repression (Table 8). Flagella-mediated motility is transcriptionally controlled in response to multiple stresses including heat-shock (RpoH dependent), pH change (CpxAR dependent), envelope stress (OmpR/EnvZ, CpxAR and PspF dependent) and low iron content (Fur dependent) (Shin and Park, 1995; Prüß et al., 2003; Lloyd et al., 2004; Jovanovic et al., 2006; Raivio et al., 2013), which are all of relevance to the conditions experienced upon EWMM exposure, as indicated by the transcriptomics data. The down-regulation of flagella/motility genes is consistent with AFM (Atomic Force Microscopy) observation in EWMM showing lack of flagella at 30 and 45°C in *E. coli* (Jan et al., 2013). Repression of motility genes at high pH (8.6) was also shown by Maurer et al. (2005), which matches the expression effects reported here. It should be stressed that some reports indicate that motility is a requirement for egg colonization by *S*. Enteritidis; non-motile *S*. Enteritidis mutants (*fliC* and *motAB*) are unable to grow in egg white or colonize eggs (Cogan et al., 2004), and a non-flagellated *flgG* mutant of *S*. Enteritidis showed a reduced survival in egg albumen (Gantois et al., 2008a). These observations suggest that the reduced expression of motility factors in EWMM, as observed here, might at least partly explain the inability of *S*. Enteritidis to propagate in this medium. The shutdown of flagella production and motility may represent an energy-conserving strategy since flagella motion is energy demanding (Zhao et al., 2007).

The reason for the induction of the *kdp* system (Table 9) is unclear because K^+^ levels in egg white medium are not limited (~36 mM in egg white; Nys and Sauveur, 2004) and the osmolarity of egg white medium (~240 mosm/L; Alabdeh, 2012) is comparable to that of the pre-culture medium, TSB (~320 mosm/L; (Alabdeh, 2012). However, KdpD kinase senses intracellular rather than extracellular K^+^ and K^+^ is crucial for the regulation of intracellular pH, as well as for the activity of several enzymes (Page and Di Cera, 2006). In the conditions used here (no extra-cellular K^+^ limitation, no osmotic shock, alkaline pH, physiological and metabolic adaptation to egg white conditions), Kdp activation probably reflects an attempt to alter cellular levels of K^+^ in response to exposure to EWMM.

#### Shift in energy metabolism and catabolism

The expression data also provide evidence for a major switch in energy-generation mode since most of the genes involved in respiration were repressed (Table 10). It is likely that their repression is largely caused by the low-iron conditions of EWMM as part of an iron-rationing response induced to reduce the demand for iron. The iron-rationing response is controlled indirectly by Fur through RfrA and RfrB in *Salmonella* (RyhB in *E. coli*) and largely functions to repress genes encoding iron-containing proteins in reaction to low iron conditions (McHugh et al., 2003; Massé et al., 2005; Yang et al., 2013). Thus, the observed down regulation of the genes listed in Table 10 appears, in part at least, to represent an attempt by *S*. Enteritidis to reprioritize cellular iron deployment. The down-regulation of these genes is likely to also include regulatory effects mediated by CpxAR (envelope and/or pH stress) and PspF (pmf dissipation). These effects correlate well with the low iron availability, high pH and membrane-disruption capacity associated with egg white (Baron et al., 2016).

The apparent loss of respiratory capacity was accompanied by other expression changes suggesting modifications in energy generation and catabolic processes (Table 11). The major induction of the 15 genes associated with hexonate and hexuronate metabolism was unexpected and is unexplained. These systems have not been previously reported to possess any role in egg white survival, nor have they been shown to be up-regulated by egg white exposure or to be subject to co-regulation. The main carbohydrate present in egg white is glucose (98% of total sugar; 0.4–0.5% w/v), with lower levels of other sugars which include mannose, galactose, arabinose, xylose, ribose and deoxyribose (Guérin-Dubiard et al., 2010). As hexonates and hexuronates are not reported to be present within egg white, the identity of the inducer (and its source) responsible for *dgoRKADT, uxuAB-uxaC* and SEN1433-6 up-regulation is unclear, although evidently these genes are not subject to any substantial catabolite repression since induction is observed despite the high glucose levels in egg white. In summary, the clear implication of these data is that during incubation in egg white, *S*. Enteritidis is exposed to hexonates and/or hexuronates, or a related compound, which results in co-induction of the *dgo, uxu* and SEN1433-6 gene sets that potentially represent a novel regulon. Since egg white lacks any of these organic acids, the most likely reason for the induction observed would appear to be the release of an endogenous inducer from *S*. Enteritidis, possibly in response to cell envelope damage. However, further experiments are required in order to clarify how EWMM exposure results in the induction of this set of genes and whether hexonate/hexuronate metabolism plays any role in egg white survival.

Increased glucose and hexonate/hexuronate catabolic capacity, increased uptake of carbohydrate and glycolysis along with serine breakdown via the One-Carbon pathway (induction of genes involved in the degradation of serine, glycine and threonine to pyruvate: *gcvHT, sdaA, glyA, kbl, tdh*; *serB*; Table 11) would be anticipated to boost ATP production through substrate-level phosphorylation which could at least partly compensate for loss of respiratory capacity and reduced expression of respiratory components.

The combined up-regulation of glycolysis with the down regulation of both the TCA cycle and respiration, as indicated above, is suggestive of a shift in energy metabolism away from respiration and toward fermentation. Indeed, the acetate kinase (*ackA*) and ethanol dehydrogenase (*adhP*) genes encoding mixed-acid fermentation enzymes are induced, supporting this notion. It is possible that the resulting accumulation of organic acids contributes to alkaline pH adaptation (Stancik et al., 2002; Yohannes et al., 2004; Slonczewski et al., 2009).

In conclusion, the gene expression response of *S*. Enteritidis to EWMM exposure at 45°C reveals three major effects: nutrient deprivation; cell damage/stress; and a shift in energy metabolism and catabolism, as summarized in Figure 2.

### Comparison of transcriptomic and mutation data

There is a general agreement that the key processes in egg white defense are iron deficiency (through iron chelation by ovotransferrin) and disruption of bacterial membranes by antimicrobial compounds (lysozyme, ovotransferrin, and various other antimicrobial molecules) (Clavijo et al., 2006; Kang et al., 2006; Baron et al., 2016). Previous mutagenesis-based studies revealed a number of specific cell functions required for *S*. Enteritidis to overcome egg white defenses (Cogan et al., 2001; Lu et al., 2003; Kang et al., 2006; Gantois et al., 2008a, 2009b; Raspoet et al., 2014). These include sugar, amino acid and nucleic acid metabolism, cell envelope biogenesis and maintenance (including LPS biosynthesis), motility, iron transport, DNA synthesis and repair, stress responses, invasion and pathogenicity (Cogan et al., 2001; Lu et al., 2003; Clavijo et al., 2006; Kang et al., 2006; Gantois et al., 2008b, 2009a; Baron et al., 2016 for review). However, such mutagenesis studies do not provide a comprehensive view of the combined and relative effects that egg white exerts upon *S*. Enteritidis since the numbers of genes identified in each individual study are limited and the experimental conditions employed are distinct for each study (Baron et al., 2016 for review). In contrast, our investigation of the global expression response of *S*. Enteritidis to egg white provides a complete genome-wide view of the complex physiological response of this pathogen to egg white-induced bactericide under specific conditions. The three major alterations in cell function (Figure 2) appear to well match the requirements of *S*. Enteritidis when challenged with egg white.

However, the gene sets showing major expression changes in the bactericidal conditions of EWMM are not a precise match for those highlighted by previous mutagenesis approaches although they do compare well with the corresponding functional-gene categories identified in earlier work, and thus the data presented here can be considered to generally support previous findings. One notable distinction is the failure to observe any modulation of genes related to LPS biosynthesis. Gantois et al. (2009b) showed that the *rfbH* gene, involved in lipopolysaccharide biosynthesis, is essential for survival at 42°C, and Raspoet et al. (2014) identified 16 lipopolysaccharide biosynthesis genes required for survival in egg white at 42°C for 24 h. This discrepancy likely relates to differences in conditions (e.g., temperature and incubation times) and experimental approaches employed, e.g., expression vs. survival.

### Insight into the bactericidal action of egg white

There are a number of bactericidal and bacteriostatic factors combined within egg white that operate together to generate a highly effective antibacterial cocktail. This amalgamation of components can be expected to exert a mutually-dependent, combinatorial antibacterial influence. Nutrient deprival should act to restrict pathogen growth and thus limit capacity to react to lethal stresses that induce envelope disruption. A key component of egg white defense, illustrated in this work, is temperature. Thus, *S*. Enteritidis suffers bacteriostasis upon exposure to egg white at moderate temperature (30°C) but is lysed at 45°C (although not in TSB) showing that the lytic activity of egg white is markedly enhanced by temperature. This observation suggests that the lethal antibacterial egg white components affecting *S*. Enteritidis survival operate optimally at ~45°C, a feature possibly related to increased bacterial membrane fluidity at higher temperature (Los and Murata, 2004).

**Figure 3:**
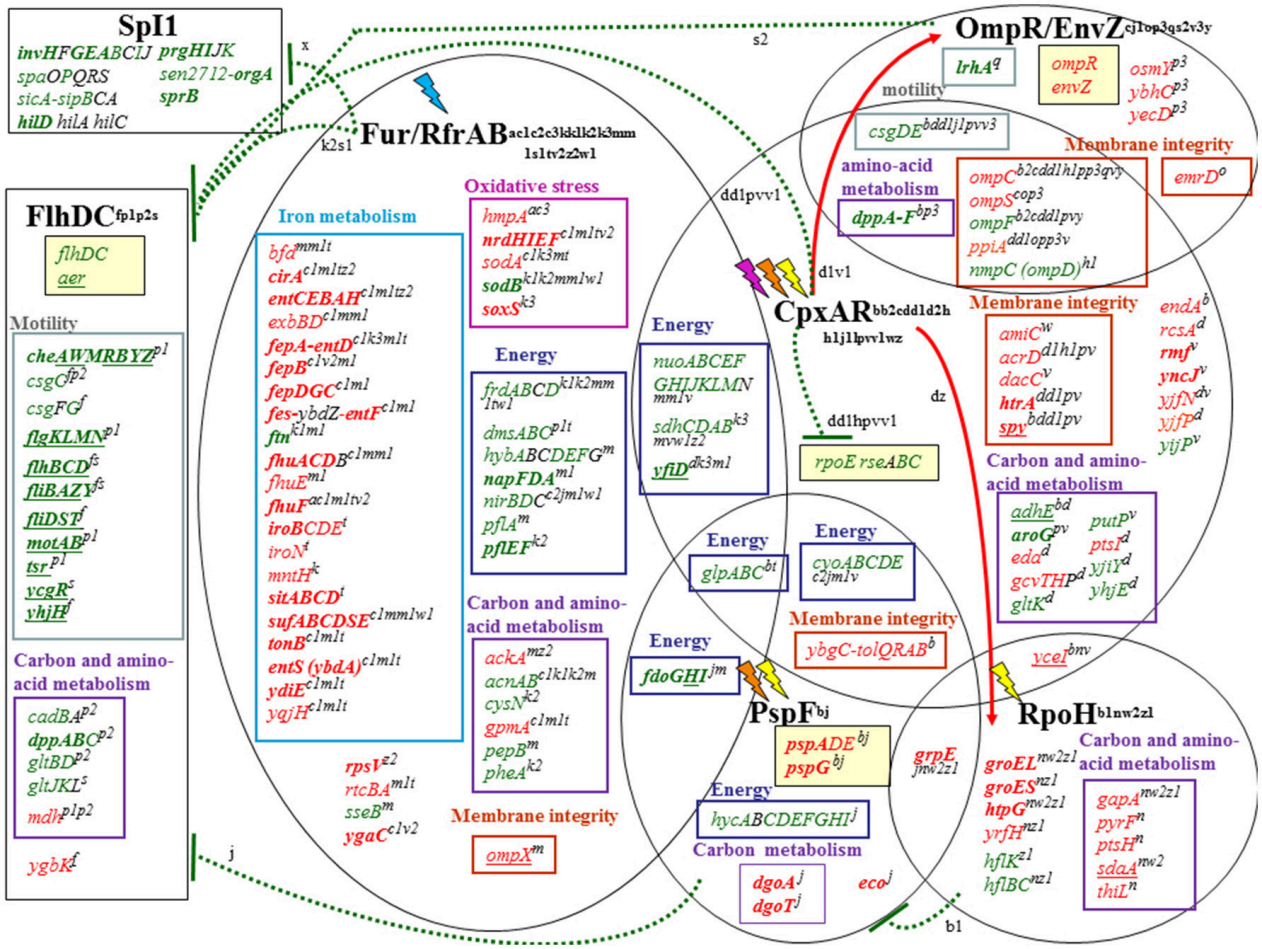
**Summary of the major global regulatory responses of ***S***. Enteritidis to bacteriocidal egg white exposure**. Each regulon is represented by black elipses and lighting symbols represent the stimuli that activate regulator responses: pink for antimicrobial molecules, orange for alkaline stress, yellow for thermal stress and blue for low iron conditions. Up-regulated, down-regulated and non-regulated genes are represented in red, green or black, respectively. Genes with a ≥fourfold change in expression are in bold. Genes underlined are identified as up or down regulated by alkaline pH (Maurer et al., 2005). Red arrows and green broken lines indicate, respectively, activation (red) and repression (green) by corresponding regulators: the origin of the arrows indicates the regulator involved. In each regulon, genes are grouped in boxes depending of the metabolic pathway to which they belong: iron metabolism (turquoise), oxidative stress (fuchsia), energy (dark blue), carbon and amino-acid metabolism (purple), motility (gray) and membrane integrity (orange). Genes previously reported to be regulated by the corresponding regulators are indicated by uppercase letters with the following designations: Brissette et al., 1991 [b1]; Stojiljkovic et al., 1994 [s1]; Crawford and Goldberg, 1998 [c3]; Danese and Silhavy, 1998 [d2]; Vassinova and Kozyrev, 2000 [v2]; Prüß et al., 2001 [p1]; D'Autréaux et al., 2002 [a]; De Wulf et al., 2002 [d]; Kehres et al., 2002 [k]; Oshima et al., 2002 [o]; McHugh et al., 2003 [m1]; Prüß et al., 2003 [p2]; Humphreys et al., 2004 [h]; Batchelor et al., 2005 [b2]; Jubelin et al., 2005 [j1]; Massé et al., 2005 [m]; Stafford et al., 2005 [s]; Vianney, 2005 [v3]; Zhao et al., 2005 [z1]; Zhang et al., 2005 [z2]; Dorel et al., 2006 [d1]; Jovanovic et al., 2006 [j]; Nonaka et al., 2006 [n]; Wade et al., 2006 [w2]; Yoshida et al., 2006 [y]; Zahrl et al., 2006 [z]; Chen et al., 2007 [c1]; Bury-Moné et al., 2009 [b]; Price and Raivio, 2009 [p]; Perkins et al., 2009 [p3]; De la Cruz and Calva, 2010 [c]; Hu et al., 2011 [h1]; Kumar and Shimizu, 2011 [k3]; Weatherspoon-Griffin et al., 2011 [w]; Troxell et al., 2011 [t]; Teixidó et al., 2011 [x]; Lin et al., 2012 [l]; Kim and Kwon, 2013a [k1]; Kim and Kwon, 2013b [k2]; Samanta et al., 2013 [s2]; Raivio et al., 2013 [v]; Wright et al., 2013 [w1]; Calderón et al., 2014 [c2]; Fitzgerald et al., 2014 [f]; Quinn et al., 2014 [q]; Raivio, 2014 [v1].

A summary of the physiological responses and major regulatory effects suggested by the transcriptional data is shown in Figures 2, 3. The regulators that appear to have the greatest influence on expression in EWMM at 45°C are Fur, RpoH, PspF, OmpR/EnvZ, CpxAR, and FlhDC. The environmental and egg white factors to which these regulators respond are low iron content (ovotransferrin mediated), unfolded proteins (induced by high temperature, alkaline pH), pmf dissipation and envelope stress (egg white membrane disruption, alkaline pH). The regulatory responses are thus an excellent representation of the environmental conditions provided by EWMM at 45°C. These regulons are highly interconnected (Figure 3). For instance, CpxAR modulates the action of various regulators including OmpR/EnvZ, FlhDC, RpoE and RpoH (Dorel et al., 2006; Raivio, 2014). Moreover, Fur, PspF and OmpR/EnvZ regulate FlhDC (Stojiljkovic and Hantke, 1992; Campoy et al., 2002; Jovanovic et al., 2006; Samanta et al., 2013) and PspF is negatively regulated by RpoH involved in the heat shock response (Brissette et al., 1991). Some of the differentially-regulated genes listed above are subject to joint control by more than one of the transcription factors highlighted in Figure 3.

The transcriptomic analysis performed here shows that incubation in EWMM at 45°C provokes a major modification of *S*. Enteritidis physiology that can be presumed to raise capacity to withstand the harmful effects imposed. However, although *S*. Enteritidis displays strong resistance to egg white, it surrcumbs to its bacteriocidal influences at temperatures of ≥42°C (Alabdeh et al., 2011). The temperature condition employed here (45°C) matches with that recommended for the French patent (Liot and Anza, 1996) that decribes a highly-efficient liquid egg white microbial stabilization process. This temperature is similar to that encountered naturally during egg formation in the hen genital tract (42°C), and during hatching (~42°C). Interestingly, a previous study (Keller et al., 1995) showed that although *S*. Enteritidis infection of the reproductive organs causes significant contamination of eggs prior to oviposition, the level of contamination is greatly decreased after laying. It was suggested that this is due to the enhanced bacteriocidal effects of egg white during the full egg formation phase in the oviduct (over 21 h), which would be promoted by the temperature of the hen oviduct (42°C). This observation provides further evidence that the precise environmental conditions of egg white are instrumental for achieving optimal antibacterial activity and that the innate immunity of the egg white is “designed” for maximum effect according to the environmental parameters that prevail during egg formation and hatching.

## Author contributions

MA: Planning and carrying out experiments, analysis and interpretation of the microarray data, drafting a part of the results sections. MC: Planning and carrying out experiments, analysis of the microarray and RT-PCR data, drafting the materials and methods and references sections. FB, SB, SJ, MG, FN, CG, SA: Conception and design of the work, design of the experiments, data interpretation, drafting parts of the results section or discussion (according to the specific specialty of each author: microbiology, molecular biology, biochemistry, iron metabolism). In addition, FB, SB, SA critically revised the entire manuscript for important intellectual content. All authors: final approval; agreement for accountability.

## Funding

MA received funding from the French Government.

### Conflict of interest statement

The authors declare that the research was conducted in the absence of any commercial or financial relationships that could be construed as a potential conflict of interest.
